# Combining mitochondrial proteomes and Mendelian randomization to identify novel therapeutic targets for diabetic nephropathy

**DOI:** 10.1080/0886022X.2025.2473669

**Published:** 2025-03-24

**Authors:** Yang Liu, Rong Wu, Zhelun Zhou, Junan Zhou, Jiaai Zhang, Xiaoyi Wang

**Affiliations:** a Center for Drug Safety Evaluation and Research, College of Pharmaceutical Sciences, Zhejiang University, Hangzhou, China; b Huzhou Key Laboratory of Chronic Kidney Disease, First Affiliated Hospital of Huzhou University, Huzhou, China

**Keywords:** Diabetic nephropathy, mitochondria, Mendelian randomization, proteomes

## Abstract

Diabetic nephropathy (DN) is a common microvascular complication of diabetes. Mitochondrial dysfunction in the kidney caused by diabetes has previously been linked to the pathogenesis of DN. By mass spectrometry, we identified characteristic proteins of DN from the renal mitochondria in mouse model. To identify the core proteins among them, Mendelian randomization (MR) analysis, microarray data validation, and drug–target interaction analysis were employed. MR analysis found that 189 candidate targets had a causal link with DN risk factors (estimated glomerular filtration rate (eGFR), urinary albumin excretion, and serum creatinine). After systematic analysis, we validated that SLC25A16, CTNND1, C2CD2L, ALDH3A2, NEU1, APEH, CORO1A, NUDT19, and NDUFA4L2 are the core proteins with promising druggability in DN. This study suggests the feasibility of using MR analysis for DN drug target screening, and provides potential insights into mitochondrial dysfunction research, which may contribute to further DN pathogenesis exploration.

## 1. Introduction

Diabetic nephropathy (DN) is a significant microvascular complication of diabetes, leading cause of chronic kidney disease (CKD) and end-stage renal disease (ESRD) [1–3]. It is characterized by persistent proteinuria, decreased glomerular filtration rate, and various pathological changes in the kidney. As the global diabetes epidemic, the incidence rate of DN also increases sharply. According to recent estimates, more than 500 million adults (20–79 years old) around the world currently suffer from diabetes, which is expected to increase gradually to 783 million by 2045 [4]. This escalating diabetes prevalence directly related to the increase of DN incidence, since up to 40% of diabetic patients will develop to CKD [5–7]. The growing incidence of DN has brought considerable pressure to the global medical system [8,9]. Renal replacement therapies, including dialysis and kidney transplantation, are costly and resource-limited. In many countries, the opportunities to obtain these therapies are limited, resulting a high mortality rate for DN patients who progress to ESRD [6–8]. Therefore, explore new therapeutic targets for facilitating early intervention to improve patient prognosis are imperative.

The pathogenesis of DN is multifaceted, involving a complex interplay of metabolic, hemodynamic, oxidative stress, inflammatory, and genetic factors [10–12]. Among them, hyperglycemia is the most important factors, capable of initiating a cascade of biochemical and molecular events leading kidney damage [12,13]. Therefore, glycemic control is the focus of DN treatment. However, some DN patients still develop to ESRD despite good glycemic control [14]. This probable due to some pathological changes have already occurred. Kidney is a high oxygen organ with mitochondrial abundance, and studies have emphasized the pivotal roles of mitochondrial pathological changes in the rapid progression of DN in early stages [15,16].

Mitochondria are the cellular powerhouses, playing a central role in the energy supply-demand balance. Under hyperglycemia conditions, this balance is disrupted, causing abnormality of mitochondrial function. This dysfunction of mitochondria in DN has been implicated in various pathophysiological processes, including glomerular hyperfiltration, basement membrane thickening, glomerulosclerosis, and tubulointerstitial fibrosis [16–18]. These changes contribute to the progressive decline in renal function, ultimately leading to ESRD. Hence, understanding the mechanisms of mitochondrial dysfunction is crucial for developing effective therapeutic strategies for DN. Many research efforts have focused on identifying molecular targets that can restore mitochondrial function. However, in the context of DN, numerous proteins undergo changes, and which of them are potential core targets for drug development has always been elusive.

Mendelian randomization (MR) is a statistical analysis of genetics that usually utilizes single nucleotide polymorphisms (SNPs) as instrumental variables (IVs) to infer causality between exposures and outcomes [19,20]. SNPs can directly alter the amino acid composition of gene-encoded proteins, and also affect protein expression by influencing protein regulatory regions. Nowadays, MR-based strategies have been used to identify potential therapeutic targets for many diseases, such as Immune diseases, cancer, and kidney disease [21–23]. In this study, we constructed a DN model in mice and identified characteristic proteins in the kidney of DN by mass spectrometry. Then use drug target MR analysis to investigate potential therapeutic targets for DN. We identify several likely causal mitochondrial proteins, report the anticipated effects on DN-related traits through renal biopsy gene expression profile, and characterize their druggability properties as potential therapeutic targets for DN.

## 2. Material and method

### 2.1. Animal model

BKS-*Lepr^em2Cd479^*/Gpt (BKS-db) mice were procured from GemPharmatech Co., Ltd. (Nanjing, China). For this study, 8-week-old BKS-db and BKS-wt (as control) were selected. Each group consists of six mice. Mice were fed with standard diet. At week 20, urines were collected using metabolic cages. Blood glucose levels were measured using a glucometer (Accu-Chek Guide Wireless Blood Glucose Meter, Roche, Basel, Switzerland). Urine protein was determined by a commercial kit (Nanjing Jiancheng Corp., Nanjing, China; C035-2-1). The mice were human sacrificed and dissected for kidney sampling.

### 2.2. Histopathology

The kidney tissues were fixed with 4% paraformaldehyde, embedded in wax blocks and cut into 5 μm paraffin sections. After deparaffinization, hematoxylin–eosin (HE) staining, periodic acid schiff (PAS) staining and Masson’s trichrome (Masson) staining were performed by stain Kits according to the manufacturer’s instructions (Beyotime, Haimen, China; C0105s, C0142s, C0189S).

### 2.3. Transmission electron microscopy

The fresh kidney tissues were sliced (1 mm) on ice and fixed with 2.5% glutaraldehyde. And then fixed with 1% osmic acid. After fixation, kidney tissues were dehydrated with an acetone gradient, embedded in epoxy resin, made into ultrathin sections, then observed the mitochondrial ultrastructure under a HITACHI HT7800 transmission electron microscopy.

### 2.4. Mitochondria proteins extract

Renal cortex was separated by dissection from freshly harvested kidneys. Mitochondria extracts were prepared using the Minute™ Mitochondrial Isolation Kit for Mammalian Cells and Tissues (invent biotechnologies Inc., Plymouth, MN, USA; MP-007) according to manufacturer’s instructions. Mitochondrial proteins were extracted from mitochondria pellets with SDC lysis buffer (5% Sodium deoxycholate, 100 mM Tris–HCl, pH 8.5).

### 2.5. Mass spectrometry assay

DTT was added to each lysate sample and react at 37 °C for 1.5 h. Iodoacetamide was then added and react at room temperature in the dark for 30 min. Trypsin (trypsin:protein (wt/wt) ratio of 1:50) was subsequently added and the mixture was incubated at 37 °C for 15 h. After desalting and concentrating, the digested peptide was reconstituted in 20 μL of 0.1% (v/v) formic acid in water. The peptide content was estimated by measuring the UV spectral density at 280 nm. For DIA experiments, the calibration retention time (iRT) calibration peptide was spiked into the sample. The sample were analyzed by Orbitrap™ Astral™ mass spectrometer (Thermo Scientific, Waltham, MA, USA) connected to an Vanquish Neo system liquid chromatography (Thermo Scientific) in the data-independent acquisition (DIA) mode. Precursor Ions were scanned at a mass range of 380–980 m/z, MS1 resolution was 240,000 at 200 m/z, Normalized AGC Target: 500%, Maximum IT: 5 ms. 299 windows were set for DIA mode in MS2 scanning, Isolation Window: 2 m/z, HCD Collision Energy: 25 ev, Normalized AGC Target: 500%, Maximum IT: 3 ms.

### 2.6. Mendelian randomization

Two-sample MR analysis was performed to explore the causal relationship between characteristic proteins identified by mass spectrometry and the DN risk factors, defining SNPs as IVs. The selection of IVs in this study should adhere to three assumptions: (1) IVs needs to be strongly correlated with exposure; (2) IVs are independent of other potential known confounding variables; (3) IVs can only affect the outcome through exposure [22–24].

We obtained SNPs of characteristic mitochondrial- protein-related genes as exposure factors and SNPs of estimated glomerular filtration rate (eGFR), urinary albumin excretion, and serum creatinine level as outcome factors from the NHGRI-EBI Catalog of human genome-wide association studies database (https://www.ebi.ac.uk/gwas/home). For the GWAS summary data on DN risk, SNP data were collected from nine datasets, including eGFR-creatinine (GCST003372, GCST90026654, and GCST90103634), urinary albumin excretion (GCST006586, GCST008794, and GCST009640) and serum creatinine level (GCST90014001, GCST90018979, and GCST90025946). The *p* value of genome-wide significance level was initially set to 5 × 10^−8^. However, we found that the number of screened SNPs was too low. To maintain genetic variation, the number, and statistical power of SNPs, we relaxed the threshold to 5 × 10^−6^, which is a commonly used threshold in many MR studies [25,26]. Then we removed SNPs that had linkage disequilibrium reactions (*r*^2 ^> 0.01, clumping distance <10,000 kb) [20,26,27]. Potential confounders were identified based on previous literature, and SNPs associated with confounding factors were identified and excluded by searching the NHGRI-EBI Catalog [20,27–29]. To test whether there was a weak IV bias, we calculated the F statistic (F = R^2^(n − *k* − 1)/k(1 − R^2^). R^2^, variance of exposure explained by selected IVs; n, sample size; and k, number of IVs. And all SNPs with F values less than 10 were removed (Supplementary Table S2).

MR analysis was performed using the ‘TwoSampleMR’ package, and the relationship between related gene expression levels and DN risk was assessed using the inverse variance weighted and Wald ratio method [24,30,31]. Heterogeneity was tested by Cochran’s Q statistic, and potential horizontal pleiotropy was evaluated using MR-Egger regression analysis. Cochran’s Q-derived *p* < 0.05 was considered as horizontal pleiotropy. MR-Egger regression intercept as an indicator (*p* < 0.05 indicates directional pleiotropy) to detect proxy IVs with directional pleiotropy. Benjamini–Hochberg procedure was performed for MR results FDR corrections [32]. When the *p*-value is greater than 0.05, there is no causal relationship between the exposure and the outcome. When the *p*-value is less than 0.05 but the FDR-adjusted *p*-value (*q*-value) is greater than 0.05, the estimated causal association is suggestive. When *q*-value is less than 0.05, there is a strong causal relationship between the exposure and the outcome. These results were shown in Supplementary Table S3–S4.

### 2.7. Microarray expression data collection and processing

The mRNA expression profiles of renal biopsy were obtained from National Center for NCBI Gene Expression Omnibus (GEO) database (https://www.ncbi.nlm.nih.gov/geo/). The search terms included ‘diabetic nephropathy’, ‘renal biopsy’, ‘kidney’, and ‘Homo sapiens’. Discarded datasets obtained from cell lines or without normal control. As last, GES104957, GSE47185, and GSE99325 datasets were included. For dataset merging, we first use the R software package inSilicoGrinding to merge the datasets [33], then *via* empirical Bayes methods to remove batch effects [34]. Control and DN samples were extracted.

### 2.8. Functional enrichment analysis

Kyoto Encyclopedia of Genes and Genomes (KEGG), Reactome and National Human Genome Research Institute (NHGRI) GWAS Catalog enrichment analysis was performed by KEGG Orthology Based Annotation System (KOBAS 3.0 http://bioinfo.org/kobas). Gene Ontology, Subcellular Localization and Human Phenotype enrichment analysis was performed by STRING database (https://cn.string-db.org/). GSVA analysis was performed using the gsva package (version 1.46.0) in software environment R (version 4.2.0). Expression values of risk and resistance dataset genes were used to perform GSVA with standard settings, the result of GSVA score was shown in Supplementary Table S6.

### 2.9. Molecular docking

The protein structures were obtained from AlphaFold Protein Structure Database (AFDB) and Protein Data Bank in Europe (PDBe). All ligand structures were downloaded from Pubchem database in SDF format. Protein structures were prepared using AutoDockTools version 1.5.7. Chain breaks were repaired when necessary, and missing side chains were modeled using the default parameters. Ligand structures were processed using Meeko, which provides more efficient and accurate ligand preparation compared to traditional AutoDockTools. The docking grid box was centered on the binding site of each protein target. The grid box dimensions were determined to cover the entire binding pocket with margins of at least 10 Å, ensuring sufficient space for ligand conformational sampling. Molecular docking simulations were performed using AutoDock Vina version 1.2.3. The binding poses were ranked according to their predicted binding affinities (kcal/mol). Molecular docking results were shown in Supplementary Table S8.

## 3. Result

### 3.1. Diabetic nephropathy model building and mitochondrial proteomes analysis

**Figure 1.**
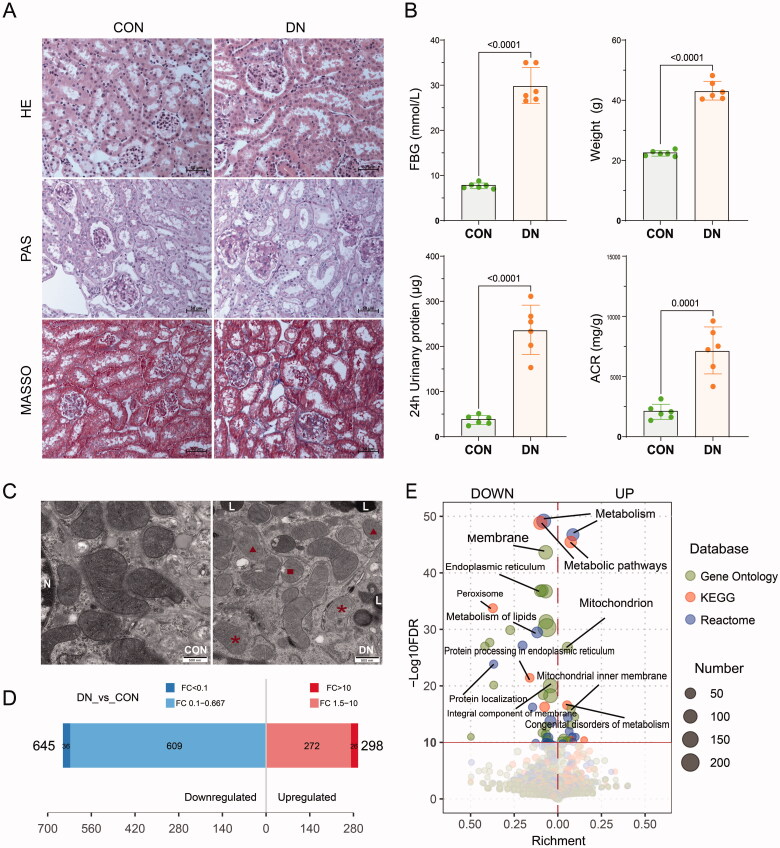
Mass spectrometric analysis of mitochondrial proteins in renal cortex of diabetic nephropathy model (DN). (A) HA, PAS, and masson staining of renal cortex. Scale bar: 500 μm. (B) Statistical results of FBG (fasting blood glucose), weight, 24-h albuminuria and urine albumin/creatinine ratio (ACR) (*n* = 6). (C) Transmission electron microscope images of renal tubular mitochondria. N: nucleus; L: lysosome; M: mitochondria; ▲: ridges disappear; ■: mitochondrial vacuole; _*:_content leakage. Scale bar: 500 nm. (D) Statistical results of identified mitochondrial proteins by mass spectrometry (*n* = 4). (E) Functional enrichment analysis of differentially expressed proteins.

To explore the characteristic mitochondrial protein in DN, we established a DN model by BKS-db mice. These mice carry gene mutations, which lead leptin receptor cannot be code normally, and cause obesity and diabetes symptoms. As our results shown, fasting blood glucose (FBG), body weight, 24 h urine protein, urine protein-to-creatinine ratio (ACR) were significantly increased in DN models compared to control in 20-week-old (Figure 1(B)). The results of HE staining shown that glomerular hypertrophy and tubular dilatation in renal cortex of DN models (Figure 1(A)). Glomerular mesangial expansion and mild renal fibrosis could also be observed in DN models by PAS and Masson’s trichrome (Masson) staining (Figure 1(A)). We next observed the ultrastructure of renal tubules mitochondria *via* transmission electron microscope. The results shown that mitochondrial ridges disappeared, vacuoles formed, and electron dense decreased in DN models (Figure 1(C)). These results suggested that the DN models were successfully established. Then we explored the characteristic mitochondrial proteins extracted from renal cortex by mass spectrometry (Figure 1(D) and Supplementary Table S1). The enrichment analysis shown that most of the differential proteins were related to metabolism, membrane, endoplasmic reticulum, and mitochondrion terms (Figure 1€).

### 3.2. Association between DN risk and characteristic mitochondrial proteins identified by MR

**Figure 2.**
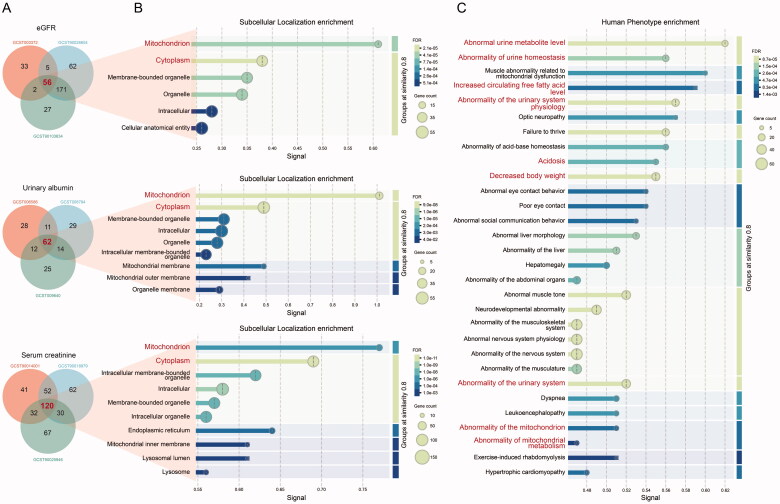
Enrichment analysis based on eGFR, urinary albumin excretion and serum creatinine levels MR results. (A) Venn diagram analyses. (B) Subcellular localization enrichment of targets identified by MR. (C) Human phenotype enrichment of all the targets identified by MR in eGFR, urinary albumin excretion, and serum creatinine datasets.

We used MR to analyze the relationship between the characteristic proteins identified by mass spectrometry and DN risk. Defining SNPs as IVs (Supplementary Table S2). Given the excessive heterogeneous of DN datasets in GWAS Catolog, we selected eGFR-creatinine, urinary albumin excretion, and serum creatinine level to represented DN risk and as outcome factors. Cochran Q statistic and MR-Egger regression were used to identify outlier variants for removal to correct potential directional horizontal pleiotropy and resolve detected heterogeneity (Supplementary Table S3). The MR analysis results were shown in Supplementary Table S4. And only when all three datasets are enriched (*p* < 0.05) can they be regarded as candidate targets (Figure 2(A)). Subcellular localization enrichment indicated that these candidate targets in three datasets all located in mitochondrion, cytoplasm, and membrane-bounded organelle (Figure 2(B)). Human phenotype enrichment analysis was performed to identify the abnormal phenotype associated with these candidate targets (189 in total, duplicates excluded). The results shown that plenty of kidney relevant phenotypes were modulated by these proteins (Figure 2(C)). ‘Abnormal urine metabolite level’ and ‘Abnormal urine homeostasis’ were the highest enrichment. ‘Increased circulating free fatty acid level’, ‘Acidosis’, and ‘Decreased body weight’ terms also be enriched. Overall, these candidate mitochondrial proteins selected by MR analysis have closely linked with kidney disease phenotype.

### 3.3. Identification of core-proteins associated with DN risk

**Figure 3.**
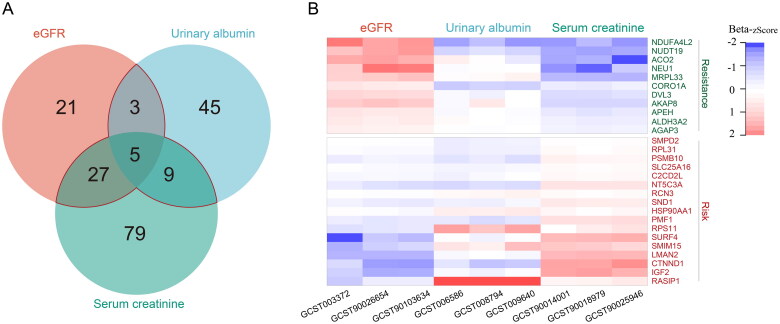
Statistical analysis of MR results. (A) Venn diagram analysis based on eGFR, urinary albumin excretion, and serum creatinine MR results. (B) Heat map of betas values estimated by MR analysis.

To further identify the core-proteins associated with DN, only candidate proteins enriched in at least two of the three datasets (eGFR, urinary albumin excretion, and serum creatinine level) can be included for the next analysis (Figure 3(A)). Betas and odds ratio (OR) with 95% confidence intervals were shown in Tables 1–2. Targets that are positively correlated with serum creatinine level or urinary albumin excretion are considered risk factors (with beta >0 and OR >1), while those negatively correlated with eGFR are also considered risk factors with the opposite direction of effect (beta <0 and OR < 1). Candidate targets in complex situations were excluded (such as target levels were positively correlated with serum creatinine, but negatively correlated with urinary albumin excretion, or positively correlated with eGFR). Table 2 shown the targets that were excluded. As a result, a total of 28 core-proteins were found (Figure 3(B), and Table 1). Among them, 17 were positively correlated with serum creatinine level or urinary albumin excretion, or negatively correlated with eGFR, were identified as risk dataset. And others were identified as resistance dataset (Figure 3(B)).

**Table 1.** Mendelian randomization analysis of the associations between candidate core proteins and DN risk factors (eGFR, urinary albumin excretion, and serum creatinine levels).

Exposure	Outcome	Nsnp	Method	beta	*p*-value	*q*-value	OR (95% CI)
ACO2	Creatinine levels (GCST90014001)	1	Wald ratio	−0.1236	<0.001	<0.001	0.884 (0.835–0.936)
	Creatinine levels (GCST90018979)	1	Wald ratio	−0.0963	0.002	0.011	0.908 (0.855–0.965)
	Creatinine levels (GCST90025946)	1	Wald ratio	−0.2074	<0.001	<0.001	0.813 (0.761–0.868)
	Glomerular filtration rate (GCST003372)	1	Wald ratio	0.0307	0.028	0.320	1.031 (1.003–1.06)
	Glomerular filtration rate (GCST90026654)	1	Wald ratio	0.0184	<0.001	<0.001	1.019 (1.011–1.027)
	Glomerular filtration rate (GCST90103634)	1	Wald ratio	0.0167	<0.001	<0.001	1.017 (1.009–1.025)
	Urinary albumin excretion (GCST006586)	1	Wald ratio	0.0153	0.586	0.845	1.015 (0.961–1.073)
	Urinary albumin excretion (GCST008794)	1	Wald ratio	0.0087	0.797	0.916	1.009 (0.944–1.078)
	Urinary albumin excretion (GCST009640)	1	Wald ratio	−0.0112	0.739	0.948	0.989 (0.926–1.056)
AGAP3	Creatinine levels (GCST90014001)	3	Inverse variance weighted	−0.0207	0.007	0.089	0.98 (0.965–0.994)
	Creatinine levels (GCST90018979)	3	Inverse variance weighted	−0.0191	0.011	0.128	0.981 (0.967–0.996)
	Creatinine levels (GCST90025946)	2	Inverse variance weighted	−0.0239	<0.001	<0.001	0.976 (0.967–0.986)
	Glomerular filtration rate (GCST003372)	2	Inverse variance weighted	0.0052	0.015	0.489	1.005 (1.001–1.009)
	Glomerular filtration rate (GCST90026654)	3	Inverse variance weighted	0.0029	<0.001	<0.001	1.003 (1.002–1.004)
	Glomerular filtration rate (GCST90103634)	3	Inverse variance weighted	0.0025	<0.001	0.004	1.002 (1.001–1.004)
	Urinary albumin excretion (GCST006586)	3	Inverse variance weighted	0.0050	0.233	0.979	1.005 (0.997–1.013)
	Urinary albumin excretion (GCST008794)	3	Inverse variance weighted	0.0039	0.430	0.937	1.004 (0.994–1.014)
	Urinary albumin excretion (GCST009640)	3	Inverse variance weighted	0.0070	0.161	0.848	1.007 (0.997–1.017)
AKAP8	Creatinine levels (GCST90014001)	1	Wald ratio	−0.0652	<0.001	0.001	0.937 (0.906–0.969)
	Creatinine levels (GCST90018979)	1	Wald ratio	−0.0550	0.003	0.018	0.946 (0.913–0.982)
	Creatinine levels (GCST90025946)	1	Wald ratio	−0.0809	<0.001	<0.001	0.922 (0.887–0.959)
	Glomerular filtration rate (GCST003372)	1	Wald ratio	0.0195	0.021	0.279	1.02 (1.003–1.037)
	Glomerular filtration rate (GCST90026654)	1	Wald ratio	0.0133	<0.001	<0.001	1.013 (1.009–1.018)
	Glomerular filtration rate (GCST90103634)	1	Wald ratio	0.0118	<0.001	<0.001	1.012 (1.007–1.017)
	Urinary albumin excretion (GCST006586)	1	Wald ratio	0.0022	0.895	0.951	1.002 (0.97–1.035)
	Urinary albumin excretion (GCST008794)	1	Wald ratio	0.0241	0.218	0.635	1.024 (0.986–1.064)
	Urinary albumin excretion (GCST009640)	1	Wald ratio	0.0051	0.794	0.968	1.005 (0.967–1.044)
ALDH3A2	Creatinine levels (GCST90014001)	1	Wald ratio	−0.0251	<0.001	0.005	0.975 (0.962–0.989)
	Creatinine levels (GCST90018979)	1	Wald ratio	−0.0221	<0.001	0.004	0.978 (0.966–0.99)
	Creatinine levels (GCST90025946)	1	Wald ratio	−0.0338	<0.001	<0.001	0.967 (0.951–0.983)
	Glomerular filtration rate (GCST003372)	1	Wald ratio	0.0082	0.017	0.247	1.008 (1.001–1.015)
	Glomerular filtration rate (GCST90026654)	1	Wald ratio	0.0039	<0.001	<0.001	1.004 (1.002–1.006)
	Glomerular filtration rate (GCST90103634)	1	Wald ratio	0.0049	<0.001	<0.001	1.005 (1.003–1.007)
	Urinary albumin excretion (GCST006586)	1	Wald ratio	0.0096	0.168	0.641	1.01 (0.996–1.024)
	Urinary albumin excretion (GCST008794)	1	Wald ratio	0.0071	0.388	0.762	1.007 (0.991–1.024)
	Urinary albumin excretion (GCST009640)	1	Wald ratio	0.0030	0.728	0.948	1.003 (0.986–1.02)
APEH	Creatinine levels (GCST90014001)	2	Inverse variance weighted	−0.0389	<0.001	<0.001	0.962 (0.951–0.973)
	Creatinine levels (GCST90018979)	2	Inverse variance weighted	−0.0404	<0.001	<0.001	0.96 (0.943–0.978)
	Creatinine levels (GCST90025946)	1	Wald ratio	−0.0473	<0.001	<0.001	0.954 (0.943–0.964)
	Glomerular filtration rate (GCST003372)	1	Wald ratio	0.0088	<0.001	0.011	1.009 (1.004–1.014)
	Glomerular filtration rate (GCST90026654)	2	Inverse variance weighted	0.0057	<0.001	<0.001	1.006 (1.003–1.008)
	Glomerular filtration rate (GCST90103634)	2	Inverse variance weighted	0.0058	<0.001	0.002	1.006 (1.003–1.009)
	Urinary albumin excretion (GCST006586)	2	Inverse variance weighted	0.0017	0.899	0.988	1.002 (0.976–1.029)
	Urinary albumin excretion (GCST008794)	2	Inverse variance weighted	0.0063	0.752	0.989	1.006 (0.968–1.046)
	Urinary albumin excretion (GCST009640)	2	Inverse variance weighted	0.0025	0.885	0.970	1.002 (0.97–1.036)
C2CD2L	Creatinine levels (GCST90014001)	2	Inverse variance weighted	0.0217	<0.001	0.003	1.022 (1.011–1.033)
	Creatinine levels (GCST90018979)	2	Inverse variance weighted	0.0207	<0.001	<0.001	1.021 (1.013–1.029)
	Creatinine levels (GCST90025946)	1	Wald ratio	0.0301	<0.001	<0.001	1.031 (1.02–1.041)
	Glomerular filtration rate (GCST003372)	1	Wald ratio	−0.0103	<0.001	0.003	0.99 (0.985–0.995)
	Glomerular filtration rate (GCST90026654)	2	Inverse variance weighted	−0.0034	<0.001	<0.001	0.997 (0.996–0.998)
	Glomerular filtration rate (GCST90103634)	2	Inverse variance weighted	−0.0039	<0.001	0.006	0.996 (0.994–0.998)
	Urinary albumin excretion (GCST006586)	2	Inverse variance weighted	−0.0032	0.595	0.988	0.997 (0.985–1.009)
	Urinary albumin excretion (GCST008794)	2	Inverse variance weighted	−0.0006	0.909	0.994	0.999 (0.99–1.009)
	Urinary albumin excretion (GCST009640)	2	Inverse variance weighted	0.0044	0.376	0.924	1.004 (0.995–1.014)
CORO1A	Creatinine levels (GCST90014001)	1	Wald ratio	−0.0322	<0.001	<0.001	0.968 (0.953–0.983)
	Creatinine levels (GCST90018979)	1	Wald ratio	−0.0288	<0.001	0.002	0.972 (0.957–0.987)
	Creatinine levels (GCST90025946)	1	Wald ratio	−0.0293	0.001	0.009	0.971 (0.954–0.989)
	Glomerular filtration rate (GCST003372)	1	Wald ratio	0.0096	0.022	0.279	1.01 (1.001–1.018)
	Glomerular filtration rate (GCST90026654)	1	Wald ratio	0.0050	<0.001	<0.001	1.005 (1.003–1.007)
	Glomerular filtration rate (GCST90103634)	1	Wald ratio	0.0046	<0.001	0.007	1.005 (1.002–1.007)
	Urinary albumin excretion (GCST006586)	1	Wald ratio	−0.0202	0.008	0.153	0.98 (0.965–0.995)
	Urinary albumin excretion (GCST008794)	1	Wald ratio	−0.0248	0.006	0.099	0.976 (0.958–0.993)
	Urinary albumin excretion (GCST009640)	1	Wald ratio	−0.0242	0.008	0.102	0.976 (0.959–0.994)
CTNND1	Creatinine levels (GCST90014001)	1	Wald ratio	0.1410	<0.001	<0.001	1.151 (1.107–1.198)
	Creatinine levels (GCST90018979)	1	Wald ratio	0.1292	<0.001	<0.001	1.138 (1.092–1.186)
	Creatinine levels (GCST90025946)	1	Wald ratio	0.1931	<0.001	<0.001	1.213 (1.159–1.27)
	Glomerular filtration rate (GCST003372)	1	Wald ratio	−0.0244	0.022	0.279	0.976 (0.956–0.997)
	Glomerular filtration rate (GCST90026654)	1	Wald ratio	−0.0212	<0.001	<0.001	0.979 (0.974–0.984)
	Glomerular filtration rate (GCST90103634)	1	Wald ratio	−0.0221	<0.001	<0.001	0.978 (0.971–0.985)
	Urinary albumin excretion (GCST006586)	1	Wald ratio	−0.0088	0.651	0.879	0.991 (0.954–1.03)
	Urinary albumin excretion (GCST008794)	1	Wald ratio	−0.0342	0.141	0.524	0.966 (0.923–1.011)
	Urinary albumin excretion (GCST009640)	1	Wald ratio	−0.0279	0.230	0.592	0.972 (0.929–1.018)
DVL3	Creatinine levels (GCST90014001)	1	Wald ratio	−0.0629	<0.001	<0.001	0.939 (0.911–0.968)
	Creatinine levels (GCST90018979)	1	Wald ratio	−0.0467	0.006	0.032	0.954 (0.923–0.987)
	Creatinine levels (GCST90025946)	1	Wald ratio	−0.0597	<0.001	0.007	0.942 (0.909–0.976)
	Glomerular filtration rate (GCST003372)	1	Wald ratio	0.0158	0.029	0.320	1.016 (1.002–1.03)
	Glomerular filtration rate (GCST90026654)	1	Wald ratio	0.0089	<0.001	<0.001	1.009 (1.005–1.013)
	Glomerular filtration rate (GCST90103634)	1	Wald ratio	0.0085	0.001	0.009	1.009 (1.003–1.014)
	Urinary albumin excretion (GCST006586)	1	Wald ratio	0.0020	0.893	0.951	1.002 (0.973–1.032)
	Urinary albumin excretion (GCST008794)	1	Wald ratio	−0.0075	0.673	0.897	0.993 (0.959–1.028)
	Urinary albumin excretion (GCST009640)	1	Wald ratio	0.0066	0.711	0.948	1.007 (0.972–1.042)
HSP90AA1	Creatinine levels (GCST90014001)	1	Wald ratio	0.0270	<0.001	<0.001	1.027 (1.015–1.04)
	Creatinine levels (GCST90018979)	1	Wald ratio	0.0158	0.004	0.020	1.016 (1.005–1.027)
	Creatinine levels (GCST90025946)	1	Wald ratio	0.0366	<0.001	<0.001	1.037 (1.023–1.052)
	Glomerular filtration rate (GCST003372)	1	Wald ratio	−0.0036	0.243	0.688	0.996 (0.99–1.002)
	Glomerular filtration rate (GCST90026654)	1	Wald ratio	−0.0025	0.001	0.008	0.998 (0.996–0.999)
	Glomerular filtration rate (GCST90103634)	1	Wald ratio	−0.0036	<0.001	0.004	0.996 (0.994–0.998)
	Urinary albumin excretion (GCST006586)	1	Wald ratio	0.0165	0.006	0.135	1.017 (1.005–1.029)
	Urinary albumin excretion (GCST008794)	1	Wald ratio	0.0191	0.008	0.117	1.019 (1.005–1.034)
	Urinary albumin excretion (GCST009640)	1	Wald ratio	0.0220	0.002	0.050	1.022 (1.008–1.037)
IGF2	Creatinine levels (GCST90014001)	1	Wald ratio	0.1389	<0.001	<0.001	1.149 (1.117–1.182)
	Creatinine levels (GCST90018979)	1	Wald ratio	0.1351	<0.001	<0.001	1.145 (1.116–1.174)
	Creatinine levels (GCST90025946)	1	Wald ratio	0.1587	<0.001	<0.001	1.172 (1.134–1.211)
	Glomerular filtration rate (GCST003372)	1	Wald ratio	−0.0279	<0.001	0.011	0.972 (0.958–0.987)
	Glomerular filtration rate (GCST90026654)	1	Wald ratio	−0.0195	<0.001	<0.001	0.981 (0.977–0.984)
	Glomerular filtration rate (GCST90103634)	1	Wald ratio	−0.0196	<0.001	<0.001	0.981 (0.976–0.985)
	Urinary albumin excretion (GCST006586)	1	Wald ratio	0.0018	0.900	0.951	1.002 (0.975–1.03)
	Urinary albumin excretion (GCST008794)	1	Wald ratio	−0.0076	0.654	0.890	0.992 (0.96–1.026)
	Urinary albumin excretion (GCST009640)	1	Wald ratio	0.0023	0.896	0.993	1.002 (0.969–1.037)
LMAN2	Creatinine levels (GCST90014001)	1	Wald ratio	0.1195	<0.001	<0.001	1.127 (1.104–1.151)
	Creatinine levels (GCST90018979)	1	Wald ratio	0.1154	<0.001	<0.001	1.122 (1.098–1.147)
	Creatinine levels (GCST90025946)	1	Wald ratio	0.1512	<0.001	<0.001	1.163 (1.136–1.191)
	Glomerular filtration rate (GCST003372)	1	Wald ratio	−0.0379	<0.001	<0.001	0.963 (0.952–0.974)
	Glomerular filtration rate (GCST90026654)	1	Wald ratio	−0.0183	<0.001	<0.001	0.982 (0.979–0.985)
	Glomerular filtration rate (GCST90103634)	1	Wald ratio	−0.0187	<0.001	<0.001	0.982 (0.978–0.985)
	Urinary albumin excretion (GCST006586)	1	Wald ratio	0.0012	0.906	0.952	1.001 (0.981–1.021)
	Urinary albumin excretion (GCST008794)	1	Wald ratio	0.0067	0.583	0.848	1.007 (0.983–1.031)
	Urinary albumin excretion (GCST009640)	1	Wald ratio	−0.0017	0.892	0.993	0.998 (0.974–1.023)
MRPL33	Creatinine levels (GCST90014001)	1	Wald ratio	−0.1074	<0.001	<0.001	0.898 (0.879–0.918)
	Creatinine levels (GCST90018979)	1	Wald ratio	−0.0907	<0.001	<0.001	0.913 (0.896–0.93)
	Creatinine levels (GCST90025946)	1	Wald ratio	−0.1332	<0.001	<0.001	0.875 (0.854–0.898)
	Glomerular filtration rate (GCST003372)	1	Wald ratio	0.0179	0.002	0.059	1.018 (1.007–1.03)
	Glomerular filtration rate (GCST90026654)	1	Wald ratio	0.0118	<0.001	<0.001	1.012 (1.009–1.015)
	Glomerular filtration rate (GCST90103634)	1	Wald ratio	0.0142	<0.001	<0.001	1.014 (1.011–1.017)
	Urinary albumin excretion (GCST006586)	1	Wald ratio	0.0085	0.431	0.803	1.009 (0.987–1.03)
	Urinary albumin excretion (GCST008794)	1	Wald ratio	0.0109	0.397	0.762	1.011 (0.986–1.037)
	Urinary albumin excretion (GCST009640)	1	Wald ratio	0.0158	0.230	0.592	1.016 (0.99–1.043)
NDUFA4L2	Creatinine levels (GCST90014001)	1	Wald ratio	−0.1366	<0.001	<0.001	0.872 (0.828–0.919)
	Creatinine levels (GCST90018979)	1	Wald ratio	−0.0855	<0.001	0.004	0.918 (0.875–0.963)
	Creatinine levels (GCST90025946)	1	Wald ratio	−0.1666	<0.001	<0.001	0.847 (0.798–0.899)
	Glomerular filtration rate (GCST003372)	1	Wald ratio	0.0397	0.005	0.112	1.04 (1.012–1.07)
	Glomerular filtration rate (GCST90026654)	1	Wald ratio	0.0180	<0.001	<0.001	1.018 (1.011–1.025)
	Glomerular filtration rate (GCST90103634)	1	Wald ratio	0.0198	<0.001	<0.001	1.02 (1.011–1.029)
	Urinary albumin excretion (GCST006586)	1	Wald ratio	−0.0328	0.200	0.678	0.968 (0.92–1.018)
	Urinary albumin excretion (GCST008794)	1	Wald ratio	−0.0354	0.245	0.656	0.965 (0.909–1.025)
	Urinary albumin excretion (GCST009640)	1	Wald ratio	−0.0473	0.121	0.498	0.954 (0.898–1.013)
NEU1	Creatinine levels (GCST90014001)	1	Wald ratio	−0.1396	<0.001	<0.001	0.87 (0.845–0.895)
	Creatinine levels (GCST90018979)	1	Wald ratio	−0.1365	<0.001	<0.001	0.872 (0.845–0.901)
	Creatinine levels (GCST90025946)	1	Wald ratio	−0.1002	0.014	0.059	0.905 (0.835–0.98)
	Glomerular filtration rate (GCST003372)	1	Wald ratio	0.0187	0.033	0.343	1.019 (1.002–1.036)
	Glomerular filtration rate (GCST90026654)	1	Wald ratio	0.0221	<0.001	<0.001	1.022 (1.018–1.027)
	Glomerular filtration rate (GCST90103634)	1	Wald ratio	0.0222	<0.001	<0.001	1.022 (1.018–1.027)
	Urinary albumin excretion (GCST006586)	1	Wald ratio	0.0030	0.833	0.940	1.003 (0.975–1.031)
	Urinary albumin excretion (GCST008794)	1	Wald ratio	0.0043	0.807	0.916	1.004 (0.97–1.039)
	Urinary albumin excretion (GCST009640)	1	Wald ratio	0.0052	0.764	0.958	1.005 (0.971–1.04)
NT5C3A	Creatinine levels (GCST90014001)	1	Wald ratio	0.0582	<0.001	<0.001	1.06 (1.039–1.082)
	Creatinine levels (GCST90018979)	1	Wald ratio	0.0565	<0.001	<0.001	1.058 (1.039–1.077)
	Creatinine levels (GCST90025946)	1	Wald ratio	0.0718	<0.001	<0.001	1.074 (1.05–1.1)
	Glomerular filtration rate (GCST003372)	1	Wald ratio	−0.0163	0.001	0.039	0.984 (0.974–0.994)
	Glomerular filtration rate (GCST90026654)	1	Wald ratio	−0.0095	<0.001	<0.001	0.991 (0.988–0.993)
	Glomerular filtration rate (GCST90103634)	1	Wald ratio	−0.0109	<0.001	<0.001	0.989 (0.986–0.992)
	Urinary albumin excretion (GCST006586)	1	Wald ratio	−0.0143	0.152	0.627	0.986 (0.967–1.005)
	Urinary albumin excretion (GCST008794)	1	Wald ratio	−0.0177	0.135	0.524	0.982 (0.96–1.006)
	Urinary albumin excretion (GCST009640)	1	Wald ratio	−0.0185	0.122	0.498	0.982 (0.959–1.005)
NUDT19	Creatinine levels (GCST90014001)	1	Wald ratio	−0.1228	<0.001	<0.001	0.884 (0.855–0.915)
	Creatinine levels (GCST90018979)	1	Wald ratio	−0.1086	<0.001	<0.001	0.897 (0.87–0.925)
	Creatinine levels (GCST90025946)	1	Wald ratio	−0.1491	<0.001	<0.001	0.862 (0.827–0.898)
	Glomerular filtration rate (GCST003372)	1	Wald ratio	0.0234	0.005	0.112	1.024 (1.007–1.04)
	Glomerular filtration rate (GCST90026654)	1	Wald ratio	0.0180	<0.001	<0.001	1.018 (1.014–1.023)
	Glomerular filtration rate (GCST90103634)	1	Wald ratio	0.0196	<0.001	<0.001	1.02 (1.014–1.026)
	Urinary albumin excretion (GCST006586)	1	Wald ratio	−0.0168	0.323	0.744	0.983 (0.951–1.017)
	Urinary albumin excretion (GCST008794)	1	Wald ratio	−0.0162	0.425	0.773	0.984 (0.946–1.024)
	Urinary albumin excretion (GCST009640)	1	Wald ratio	−0.0226	0.267	0.657	0.978 (0.939–1.017)
PMF1	Creatinine levels (GCST90014001)	1	Wald ratio	0.0699	<0.001	<0.001	1.072 (1.049–1.096)
	Creatinine levels (GCST90018979)	1	Wald ratio	0.0643	<0.001	<0.001	1.066 (1.044–1.09)
	Creatinine levels (GCST90025946)	1	Wald ratio	0.0899	<0.001	<0.001	1.094 (1.068–1.121)
	Glomerular filtration rate (GCST003372)	1	Wald ratio	−0.0153	0.007	0.129	0.985 (0.974–0.996)
	Glomerular filtration rate (GCST90026654)	1	Wald ratio	−0.0088	<0.001	<0.001	0.991 (0.988–0.994)
	Glomerular filtration rate (GCST90103634)	1	Wald ratio	−0.0080	<0.001	<0.001	0.992 (0.989–0.996)
	Urinary albumin excretion (GCST006586)	1	Wald ratio	−0.0067	0.538	0.840	0.993 (0.973–1.015)
	Urinary albumin excretion (GCST008794)	1	Wald ratio	−0.0219	0.089	0.425	0.978 (0.954–1.003)
	Urinary albumin excretion (GCST009640)	1	Wald ratio	−0.0135	0.295	0.688	0.987 (0.962–1.012)
PSMB10	Creatinine levels (GCST90014001)	1	Wald ratio	0.0358	0.002	0.013	1.036 (1.013–1.06)
	Creatinine levels (GCST90018979)	1	Wald ratio	0.0318	0.013	0.056	1.032 (1.007–1.059)
	Creatinine levels (GCST90025946)	1	Wald ratio	0.0362	0.008	0.040	1.037 (1.009–1.065)
	Glomerular filtration rate (GCST003372)	1	Wald ratio	−0.0155	0.008	0.153	0.985 (0.973–0.996)
	Glomerular filtration rate (GCST90026654)	1	Wald ratio	−0.0047	0.003	0.014	0.995 (0.992–0.998)
	Glomerular filtration rate (GCST90103634)	1	Wald ratio	−0.0055	0.002	0.011	0.994 (0.991–0.998)
	Urinary albumin excretion (GCST006586)	1	Wald ratio	−0.0095	0.394	0.794	0.991 (0.969–1.012)
	Urinary albumin excretion (GCST008794)	1	Wald ratio	−0.0105	0.437	0.774	0.99 (0.964–1.016)
	Urinary albumin excretion (GCST009640)	1	Wald ratio	−0.0175	0.192	0.544	0.983 (0.957–1.009)
RASIP1	Creatinine levels (GCST90014001)	1	Wald ratio	0.0302	0.034	0.117	1.031 (1.002–1.06)
	Creatinine levels (GCST90018979)	1	Wald ratio	0.0364	0.021	0.077	1.037 (1.006–1.069)
	Creatinine levels (GCST90025946)	1	Wald ratio	0.0619	<0.001	0.002	1.064 (1.03–1.099)
	Glomerular filtration rate (GCST003372)	1	Wald ratio	−0.0306	<0.001	0.004	0.97 (0.956–0.984)
	Glomerular filtration rate (GCST90026654)	1	Wald ratio	−0.0066	<0.001	0.006	0.993 (0.99–0.997)
	Glomerular filtration rate (GCST90103634)	1	Wald ratio	−0.0066	0.008	0.034	0.993 (0.989–0.998)
	Urinary albumin excretion (GCST006586)	1	Wald ratio	0.0727	<0.001	<0.001	1.075 (1.047–1.105)
	Urinary albumin excretion (GCST008794)	1	Wald ratio	0.1234	<0.001	<0.001	1.131 (1.095–1.169)
	Urinary albumin excretion (GCST009640)	1	Wald ratio	0.1116	<0.001	<0.001	1.118 (1.082–1.155)
RCN3	Creatinine levels (GCST90014001)	2	Inverse variance weighted	0.0184	<0.001	0.004	1.019 (1.009–1.028)
	Creatinine levels (GCST90018979)	2	Inverse variance weighted	0.0185	0.026	0.216	1.019 (1.002–1.036)
	Creatinine levels (GCST90025946)	2	Inverse variance weighted	0.0224	0.002	0.023	1.023 (1.008–1.038)
	Glomerular filtration rate (GCST90026654)	2	Inverse variance weighted	−0.0022	0.077	0.365	0.998 (0.995–1)
	Glomerular filtration rate (GCST90103634)	2	Inverse variance weighted	−0.0022	0.069	0.409	0.998 (0.995–1)
	Urinary albumin excretion (GCST006586)	2	Inverse variance weighted	0.0101	0.006	0.268	1.01 (1.003–1.017)
	Urinary albumin excretion (GCST008794)	2	Inverse variance weighted	0.0145	0.001	0.109	1.015 (1.006–1.024)
	Urinary albumin excretion (GCST009640)	2	Inverse variance weighted	0.0161	<0.001	0.043	1.016 (1.007–1.025)
RPL31	Creatinine levels (GCST90014001)	2	Inverse variance weighted	0.0196	<0.001	0.003	1.02 (1.01–1.03)
	Creatinine levels (GCST90018979)	2	Inverse variance weighted	0.0109	0.019	0.177	1.011 (1.002–1.02)
	Creatinine levels (GCST90025946)	2	Inverse variance weighted	0.0267	<0.001	<0.001	1.027 (1.016–1.038)
	Glomerular filtration rate (GCST003372)	2	Inverse variance weighted	−0.0069	0.004	0.223	0.993 (0.989–0.998)
	Glomerular filtration rate (GCST90026654)	2	Inverse variance weighted	−0.0028	<0.001	<0.001	0.997 (0.996–0.998)
	Glomerular filtration rate (GCST90103634)	2	Inverse variance weighted	−0.0030	<0.001	0.003	0.997 (0.995–0.998)
	Urinary albumin excretion (GCST006586)	2	Inverse variance weighted	−0.0050	0.285	0.979	0.995 (0.986–1.004)
	Urinary albumin excretion (GCST008794)	2	Inverse variance weighted	−0.0065	0.241	0.853	0.994 (0.983–1.004)
	Urinary albumin excretion (GCST009640)	2	Inverse variance weighted	−0.0061	0.279	0.908	0.994 (0.983–1.005)
RPS11	Creatinine levels (GCST90018979)	1	Wald ratio	0.0451	0.002	0.010	1.046 (1.017–1.076)
	Creatinine levels (GCST90025946)	1	Wald ratio	0.0780	<0.001	<0.001	1.081 (1.047–1.116)
	Glomerular filtration rate (GCST003372)	1	Wald ratio	−0.0221	0.001	0.042	0.978 (0.965–0.991)
	Glomerular filtration rate (GCST90026654)	1	Wald ratio	−0.0084	<0.001	<0.001	0.992 (0.988–0.995)
	Glomerular filtration rate (GCST90103634)	1	Wald ratio	−0.0078	0.001	0.010	0.992 (0.987–0.997)
	Urinary albumin excretion (GCST006586)	1	Wald ratio	0.0454	0.002	0.080	1.046 (1.017–1.077)
	Urinary albumin excretion (GCST008794)	1	Wald ratio	0.0448	0.009	0.124	1.046 (1.011–1.082)
	Urinary albumin excretion (GCST009640)	1	Wald ratio	0.0554	0.002	0.050	1.057 (1.021–1.094)
SLC25A16	Creatinine levels (GCST90014001)	2	Inverse variance weighted	0.0197	<0.001	<0.001	1.02 (1.012–1.028)
	Creatinine levels (GCST90018979)	3	Inverse variance weighted	0.0192	<0.001	<0.001	1.019 (1.013–1.026)
	Creatinine levels (GCST90025946)	2	Inverse variance weighted	0.0260	<0.001	<0.001	1.026 (1.018–1.035)
	Glomerular filtration rate (GCST003372)	2	Inverse variance weighted	−0.0061	<0.001	0.068	0.994 (0.99–0.997)
	Glomerular filtration rate (GCST90026654)	3	Inverse variance weighted	−0.0032	<0.001	<0.001	0.997 (0.996–0.998)
	Glomerular filtration rate (GCST90103634)	3	Inverse variance weighted	−0.0035	<0.001	<0.001	0.996 (0.995–0.998)
	Urinary albumin excretion (GCST006586)	3	Inverse variance weighted	−0.0006	0.894	0.988	0.999 (0.991–1.008)
	Urinary albumin excretion (GCST008794)	2	Inverse variance weighted	−0.0031	0.592	0.961	0.997 (0.986–1.008)
	Urinary albumin excretion (GCST009640)	2	Inverse variance weighted	−0.0002	0.976	0.992	1 (0.99–1.01)
SMIM15	Creatinine levels (GCST90014001)	1	Wald ratio	0.1007	<0.001	0.001	1.106 (1.05–1.164)
	Creatinine levels (GCST90018979)	1	Wald ratio	0.0905	<0.001	0.006	1.095 (1.038–1.155)
	Creatinine levels (GCST90025946)	1	Wald ratio	0.1013	<0.001	0.006	1.107 (1.043–1.174)
	Glomerular filtration rate (GCST003372)	1	Wald ratio	−0.0392	0.005	0.112	0.962 (0.936–0.988)
	Glomerular filtration rate (GCST90026654)	1	Wald ratio	−0.0136	<0.001	0.001	0.986 (0.98–0.993)
	Glomerular filtration rate (GCST90103634)	1	Wald ratio	−0.0121	0.008	0.034	0.988 (0.979–0.997)
	Urinary albumin excretion (GCST006586)	1	Wald ratio	0.0203	0.423	0.803	1.02 (0.971–1.072)
	Urinary albumin excretion (GCST008794)	1	Wald ratio	0.0188	0.533	0.828	1.019 (0.96–1.081)
	Urinary albumin excretion (GCST009640)	1	Wald ratio	0.0453	0.134	0.498	1.046 (0.986–1.11)
SMPD2	Creatinine levels (GCST90014001)	1	Wald ratio	0.0110	0.003	0.018	1.011 (1.004–1.018)
	Creatinine levels (GCST90018979)	2	Inverse variance weighted	0.0081	0.048	0.308	1.008 (1–1.016)
	Creatinine levels (GCST90025946)	1	Wald ratio	0.0202	<0.001	<0.001	1.02 (1.012–1.029)
	Glomerular filtration rate (GCST003372)	1	Wald ratio	−0.0056	0.003	0.094	0.994 (0.991–0.998)
	Glomerular filtration rate (GCST90026654)	2	Inverse variance weighted	−0.0019	<0.001	0.002	0.998 (0.997–0.999)
	Glomerular filtration rate (GCST90103634)	2	Inverse variance weighted	−0.0020	0.001	0.021	0.998 (0.997–0.999)
	Urinary albumin excretion (GCST006586)	2	Inverse variance weighted	−0.0056	0.161	0.904	0.994 (0.987–1.002)
	Urinary albumin excretion (GCST008794)	2	Inverse variance weighted	−0.0073	0.094	0.698	0.993 (0.984–1.001)
	Urinary albumin excretion (GCST009640)	2	Inverse variance weighted	−0.0041	0.345	0.920	0.996 (0.987–1.004)
SND1	Creatinine levels (GCST90014001)	1	Wald ratio	0.0515	<0.001	<0.001	1.053 (1.04–1.066)
	Creatinine levels (GCST90018979)	2	Inverse variance weighted	0.0478	<0.001	<0.001	1.049 (1.035–1.063)
	Creatinine levels (GCST90025946)	1	Wald ratio	0.0582	<0.001	<0.001	1.06 (1.044–1.076)
	Glomerular filtration rate (GCST003372)	1	Wald ratio	−0.0123	<0.001	0.006	0.988 (0.982–0.994)
	Glomerular filtration rate (GCST90026654)	2	Inverse variance weighted	−0.0074	<0.001	<0.001	0.993 (0.991–0.994)
	Glomerular filtration rate (GCST90103634)	2	Inverse variance weighted	−0.0078	<0.001	<0.001	0.992 (0.99–0.994)
	Urinary albumin excretion (GCST006586)	2	Inverse variance weighted	−0.0028	0.647	0.988	0.997 (0.985–1.009)
	Urinary albumin excretion (GCST008794)	2	Inverse variance weighted	−0.0079	0.275	0.863	0.992 (0.978–1.006)
	Urinary albumin excretion (GCST009640)	2	Inverse variance weighted	−0.0007	0.929	0.977	0.999 (0.985–1.014)
SURF4	Creatinine levels (GCST90014001)	1	Wald ratio	0.1186	<0.001	<0.001	1.126 (1.062–1.194)
	Creatinine levels (GCST90018979)	1	Wald ratio	0.1131	<0.001	<0.001	1.12 (1.071–1.171)
	Creatinine levels (GCST90025946)	1	Wald ratio	0.1610	<0.001	<0.001	1.175 (1.098–1.256)
	Glomerular filtration rate (GCST003372)	1	Wald ratio	−0.0574	<0.001	0.003	0.944 (0.919–0.97)
	Glomerular filtration rate (GCST90026654)	1	Wald ratio	−0.0148	<0.001	<0.001	0.985 (0.979–0.992)
	Glomerular filtration rate (GCST90103634)	1	Wald ratio	−0.0178	<0.001	<0.001	0.982 (0.975–0.99)
	Urinary albumin excretion (GCST006586)	1	Wald ratio	0.0073	0.799	0.940	1.007 (0.952–1.066)
	Urinary albumin excretion (GCST008794)	1	Wald ratio	−0.0053	0.873	0.940	0.995 (0.932–1.062)
	Urinary albumin excretion (GCST009640)	1	Wald ratio	0.0040	0.907	0.993	1.004 (0.938–1.075)

**Table 2.** Mendelian randomization analysis of the associations between the excluded candidate proteins and DN risk factors (eGFR, urinary albumin excretion, and serum creatinine levels).

Exposure	Outcome	Nsnp	Method	beta	*p*-value	*q*-value	OR (95% CI)
AKR1A1	Creatinine levels (GCST90014001)	1	Wald ratio	0.0515	<0.001	<0.001	1.053 (1.028–1.078)
	Creatinine levels (GCST90018979)	1	Wald ratio	0.0483	<0.001	<0.001	1.049 (1.027–1.072)
	Creatinine levels (GCST90025946)	1	Wald ratio	0.0690	<0.001	<0.001	1.071 (1.043–1.101)
	Glomerular filtration rate (GCST003372)	1	Wald ratio	−0.0055	0.385	0.754	0.994 (0.982–1.007)
	Glomerular filtration rate (GCST90026654)	1	Wald ratio	−0.0081	<0.001	<0.001	0.992 (0.989–0.995)
	Glomerular filtration rate (GCST90103634)	1	Wald ratio	−0.0083	<0.001	<0.001	0.992 (0.988–0.996)
	Urinary albumin excretion (GCST006586)	1	Wald ratio	−0.0322	0.006	0.135	0.968 (0.947–0.991)
	Urinary albumin excretion (GCST008794)	1	Wald ratio	−0.0491	<0.001	0.019	0.952 (0.926–0.979)
	Urinary albumin excretion (GCST009640)	1	Wald ratio	−0.0414	0.003	0.050	0.959 (0.934–0.986)
CISD2	Creatinine levels (GCST90014001)	1	Wald ratio	0.0732	<0.001	<0.001	1.076 (1.063–1.09)
	Creatinine levels (GCST90018979)	1	Wald ratio	0.0664	<0.001	<0.001	1.069 (1.056–1.081)
	Creatinine levels (GCST90025946)	1	Wald ratio	0.0897	<0.001	<0.001	1.094 (1.078–1.11)
	Glomerular filtration rate (GCST003372)	1	Wald ratio	−0.0137	<0.001	0.004	0.986 (0.98–0.993)
	Glomerular filtration rate (GCST90026654)	1	Wald ratio	−0.0097	<0.001	<0.001	0.99 (0.989–0.992)
	Glomerular filtration rate (GCST90103634)	1	Wald ratio	−0.0104	<0.001	<0.001	0.99 (0.988–0.992)
	Urinary albumin excretion (GCST006586)	1	Wald ratio	0.0035	0.567	0.840	1.004 (0.991–1.016)
	Urinary albumin excretion (GCST008794)	1	Wald ratio	−0.0396	0.044	0.305	0.961 (0.925–0.999)
CNOT2	Creatinine levels (GCST90014001)	1	Wald ratio	0.0509	0.002	0.013	1.052 (1.019–1.087)
	Creatinine levels (GCST90018979)	1	Wald ratio	0.0416	0.025	0.087	1.042 (1.005–1.081)
	Creatinine levels (GCST90025946)	1	Wald ratio	0.0498	0.012	0.052	1.051 (1.011–1.092)
	Glomerular filtration rate (GCST003372)	1	Wald ratio	−0.0121	0.113	0.516	0.988 (0.973–1.003)
	Glomerular filtration rate (GCST90026654)	1	Wald ratio	−0.0053	0.025	0.082	0.995 (0.99–0.999)
	Glomerular filtration rate (GCST90103634)	1	Wald ratio	−0.0058	0.024	0.088	0.994 (0.989–0.999)
	Urinary albumin excretion (GCST006586)	1	Wald ratio	−0.0637	<0.001	0.006	0.938 (0.909–0.968)
	Urinary albumin excretion (GCST008794)	1	Wald ratio	−0.0710	<0.001	0.008	0.931 (0.898–0.967)
	Urinary albumin excretion (GCST009640)	1	Wald ratio	−0.0722	<0.001	0.011	0.93 (0.896–0.966)
CNPY3	Creatinine levels (GCST90014001)	1	Wald ratio	−0.0394	<0.001	<0.001	0.961 (0.945–0.978)
	Creatinine levels (GCST90018979)	1	Wald ratio	−0.0380	<0.001	<0.001	0.963 (0.945–0.98)
	Creatinine levels (GCST90025946)	1	Wald ratio	−0.0369	<0.001	0.003	0.964 (0.945–0.983)
	Glomerular filtration rate (GCST003372)	1	Wald ratio	0.0082	0.082	0.450	1.008 (0.999–1.018)
	Glomerular filtration rate (GCST90026654)	1	Wald ratio	0.0042	<0.001	0.003	1.004 (1.002–1.007)
	Glomerular filtration rate (GCST90103634)	1	Wald ratio	0.0041	0.008	0.034	1.004 (1.001–1.007)
	Urinary albumin excretion (GCST006586)	1	Wald ratio	0.0275	0.001	0.078	1.028 (1.011–1.045)
	Urinary albumin excretion (GCST008794)	1	Wald ratio	0.0341	0.001	0.033	1.035 (1.014–1.056)
	Urinary albumin excretion (GCST009640)	1	Wald ratio	0.0436	<0.001	0.002	1.045 (1.024–1.066)
CYP27A1	Creatinine levels (GCST90014001)	2	Inverse variance weighted	−0.0091	<0.001	0.004	0.991 (0.986–0.996)
	Creatinine levels (GCST90018979)	2	Inverse variance weighted	−0.0104	<0.001	<0.001	0.99 (0.986–0.993)
	Creatinine levels (GCST90025946)	2	Inverse variance weighted	−0.0115	<0.001	<0.001	0.989 (0.984–0.993)
	Glomerular filtration rate (GCST003372)	2	Inverse variance weighted	0.0025	0.046	0.509	1.003 (1–1.005)
	Glomerular filtration rate (GCST90026654)	2	Inverse variance weighted	0.0018	<0.001	<0.001	1.002 (1.001–1.002)
	Glomerular filtration rate (GCST90103634)	2	Inverse variance weighted	0.0018	<0.001	<0.001	1.002 (1.001–1.002)
	Urinary albumin excretion (GCST006586)	2	Inverse variance weighted	0.0042	0.018	0.448	1.004 (1.001–1.008)
	Urinary albumin excretion (GCST008794)	2	Inverse variance weighted	0.0071	<0.001	0.083	1.007 (1.003–1.011)
	Urinary albumin excretion (GCST009640)	2	Inverse variance weighted	0.0071	0.001	0.058	1.007 (1.003–1.011)
ERP29	Creatinine levels (GCST90014001)	1	Wald ratio	0.0451	<0.001	<0.001	1.046 (1.026–1.067)
	Creatinine levels (GCST90018979)	1	Wald ratio	0.0545	<0.001	<0.001	1.056 (1.039–1.073)
	Creatinine levels (GCST90025946)	1	Wald ratio	0.0610	<0.001	<0.001	1.063 (1.039–1.087)
	Glomerular filtration rate (GCST003372)	1	Wald ratio	−0.0127	0.011	0.178	0.987 (0.978–0.997)
	Glomerular filtration rate (GCST90026654)	1	Wald ratio	−0.0072	<0.001	<0.001	0.993 (0.99–0.995)
	Glomerular filtration rate (GCST90103634)	1	Wald ratio	−0.0077	<0.001	<0.001	0.992 (0.989–0.996)
	Urinary albumin excretion (GCST006586)	1	Wald ratio	−0.0220	0.021	0.215	0.978 (0.96–0.997)
	Urinary albumin excretion (GCST008794)	1	Wald ratio	−0.0135	0.232	0.652	0.987 (0.965–1.009)
	Urinary albumin excretion (GCST009640)	1	Wald ratio	−0.0261	0.026	0.229	0.974 (0.952–0.997)
FUCA1	Creatinine levels (GCST90014001)	8	Inverse variance weighted	0.0179	<0.001	<0.001	1.018 (1.011–1.026)
	Creatinine levels (GCST90014001)	8	Simple mode	0.0181	0.051	0.996	1.018 (1.003–1.034)
	Creatinine levels (GCST90014001)	8	Weighted median	0.0188	<0.001	<0.001	1.019 (1.01–1.028)
	Creatinine levels (GCST90014001)	8	Weighted mode	0.0190	0.003	0.720	1.019 (1.011–1.028)
	Creatinine levels (GCST90014001)	8	MR Egger	0.0197	0.175	0.999	1.02 (0.995–1.046)
	Creatinine levels (GCST90018979)	7	MR Egger	0.0007	0.956	0.998	1.001 (0.977–1.025)
	Creatinine levels (GCST90018979)	7	Weighted median	0.0157	<0.001	<0.001	1.016 (1.008–1.023)
	Creatinine levels (GCST90018979)	7	Weighted mode	0.0160	0.005	0.749	1.016 (1.009–1.024)
	Creatinine levels (GCST90018979)	7	Inverse variance weighted	0.0174	<0.001	<0.001	1.018 (1.011–1.025)
	Creatinine levels (GCST90018979)	7	Simple mode	0.0200	0.038	0.967	1.02 (1.005–1.035)
	Creatinine levels (GCST90025946)	1	Wald ratio	0.0162	0.002	0.011	1.016 (1.006–1.027)
	Glomerular filtration rate (GCST003372)	1	Wald ratio	−0.0057	0.009	0.163	0.994 (0.99–0.999)
	Glomerular filtration rate (GCST90026654)	8	Weighted median	−0.0017	0.002	0.030	0.998 (0.997–0.999)
	Glomerular filtration rate (GCST90026654)	8	Inverse variance weighted	−0.0017	<0.001	0.015	0.998 (0.997–0.999)
	Glomerular filtration rate (GCST90026654)	8	Weighted mode	−0.0016	0.022	0.662	0.998 (0.997–0.999)
	Glomerular filtration rate (GCST90026654)	8	MR Egger	−0.0015	0.458	0.992	0.999 (0.995–1.002)
	Glomerular filtration rate (GCST90026654)	8	Simple mode	−0.0012	0.277	0.940	0.999 (0.997–1.001)
	Glomerular filtration rate (GCST90103634)	8	Weighted median	−0.0024	<0.001	0.007	0.998 (0.996–0.999)
	Glomerular filtration rate (GCST90103634)	8	MR Egger	−0.0023	0.294	0.992	0.998 (0.994–1.002)
	Glomerular filtration rate (GCST90103634)	8	Inverse variance weighted	−0.0023	<0.001	0.005	0.998 (0.997–0.999)
	Glomerular filtration rate (GCST90103634)	8	Weighted mode	−0.0022	0.020	0.849	0.998 (0.996–0.999)
	Glomerular filtration rate (GCST90103634)	8	Simple mode	−0.0016	0.211	0.943	0.998 (0.996–1.001)
	Urinary albumin excretion (GCST006586)	8	Inverse variance weighted	−0.0122	0.025	0.477	0.988 (0.977–0.999)
	Urinary albumin excretion (GCST006586)	8	Weighted median	−0.0099	0.023	0.426	0.99 (0.982–0.999)
	Urinary albumin excretion (GCST006586)	8	Weighted mode	−0.0094	0.073	0.991	0.991 (0.982–0.999)
	Urinary albumin excretion (GCST006586)	8	Simple mode	−0.0058	0.643	0.998	0.994 (0.971–1.018)
	Urinary albumin excretion (GCST006586)	8	MR Egger	−0.0043	0.829	0.988	0.996 (0.959–1.034)
	Urinary albumin excretion (GCST008794)	8	Inverse variance weighted	−0.0123	0.071	0.655	0.988 (0.975–1.001)
	Urinary albumin excretion (GCST008794)	8	Weighted median	−0.0071	0.163	0.667	0.993 (0.983–1.003)
	Urinary albumin excretion (GCST008794)	8	Weighted mode	−0.0056	0.324	0.982	0.994 (0.984–1.005)
	Urinary albumin excretion (GCST008794)	8	Simple mode	0.0013	0.921	0.995	1.001 (0.977–1.026)
	Urinary albumin excretion (GCST008794)	8	MR Egger	0.0128	0.582	0.996	1.013 (0.97–1.058)
	Urinary albumin excretion (GCST009640)	8	Inverse variance weighted	−0.0127	0.020	0.443	0.987 (0.977–0.998)
	Urinary albumin excretion (GCST009640)	8	Weighted mode	−0.0104	0.084	0.989	0.99 (0.98–1)
	Urinary albumin excretion (GCST009640)	8	Weighted median	−0.0100	0.045	0.570	0.99 (0.98–1)
	Urinary albumin excretion (GCST009640)	8	MR Egger	−0.0012	0.952	0.985	0.999 (0.963–1.036)
	Urinary albumin excretion (GCST009640)	8	Simple mode	0.0001	0.993	0.996	1 (0.978–1.023)
HADHA	Creatinine levels (GCST90014001)	2	Inverse variance weighted	−0.0201	<0.001	0.003	0.98 (0.97–0.99)
	Creatinine levels (GCST90018979)	2	Inverse variance weighted	−0.0119	0.015	0.156	0.988 (0.979–0.998)
	Creatinine levels (GCST90025946)	2	Inverse variance weighted	0.0152	0.030	0.153	1.015 (1.001–1.029)
	Glomerular filtration rate (GCST003372)	2	Inverse variance weighted	−0.0012	0.642	0.988	0.999 (0.994–1.004)
	Glomerular filtration rate (GCST90026654)	2	Inverse variance weighted	0.0019	0.004	0.048	1.002 (1.001–1.003)
	Glomerular filtration rate (GCST90103634)	2	Inverse variance weighted	0.0020	0.111	0.513	1.002 (1–1.004)
	Urinary albumin excretion (GCST006586)	2	Inverse variance weighted	0.0122	0.012	0.337	1.012 (1.003–1.022)
	Urinary albumin excretion (GCST008794)	2	Inverse variance weighted	0.0151	0.036	0.510	1.015 (1.001–1.03)
	Urinary albumin excretion (GCST009640)	2	Inverse variance weighted	0.0170	0.031	0.534	1.017 (1.002–1.033)
NIF3L1	Creatinine levels (GCST90014001)	1	Wald ratio	−0.0222	0.109	0.268	0.978 (0.952–1.005)
	Creatinine levels (GCST90018979)	1	Wald ratio	−0.0367	0.017	0.066	0.964 (0.935–0.993)
	Creatinine levels (GCST90025946)	1	Wald ratio	−0.0293	0.069	0.183	0.971 (0.941–1.002)
	Glomerular filtration rate (GCST003372)	1	Wald ratio	0.0140	0.036	0.367	1.014 (1.001–1.027)
	Glomerular filtration rate (GCST90026654)	1	Wald ratio	0.0037	0.049	0.129	1.004 (1–1.007)
	Glomerular filtration rate (GCST90103634)	1	Wald ratio	0.0040	0.046	0.134	1.004 (1–1.008)
	Urinary albumin excretion (GCST006586)	1	Wald ratio	0.0346	0.010	0.163	1.035 (1.008–1.063)
	Urinary albumin excretion (GCST008794)	1	Wald ratio	0.0363	0.022	0.206	1.037 (1.005–1.07)
	Urinary albumin excretion (GCST009640)	1	Wald ratio	0.0474	0.003	0.051	1.049 (1.016–1.082)
NT5DC2	Creatinine levels (GCST90014001)	2	Inverse variance weighted	0.0312	0.003	0.051	1.032 (1.01–1.053)
	Creatinine levels (GCST90018979)	2	Inverse variance weighted	0.0210	<0.001	0.007	1.021 (1.01–1.033)
	Creatinine levels (GCST90025946)	1	Wald ratio	0.0440	<0.001	<0.001	1.045 (1.035–1.055)
	Glomerular filtration rate (GCST003372)	1	Wald ratio	−0.0071	0.001	0.039	0.993 (0.989–0.997)
	Glomerular filtration rate (GCST90026654)	2	Inverse variance weighted	−0.0036	0.001	0.021	0.996 (0.994–0.999)
	Glomerular filtration rate (GCST90103634)	2	Inverse variance weighted	−0.0042	0.006	0.080	0.996 (0.993–0.999)
	Urinary albumin excretion (GCST006586)	2	Inverse variance weighted	−0.0160	0.021	0.450	0.984 (0.971–0.998)
	Urinary albumin excretion (GCST008794)	2	Inverse variance weighted	−0.0234	<0.001	0.027	0.977 (0.965–0.989)
	Urinary albumin excretion (GCST009640)	2	Inverse variance weighted	−0.0200	0.050	0.603	0.98 (0.961–1)
P4HA1	Creatinine levels (GCST90014001)	1	Wald ratio	0.0519	<0.001	0.001	1.053 (1.026–1.081)
	Creatinine levels (GCST90018979)	1	Wald ratio	0.0481	<0.001	<0.001	1.049 (1.03–1.069)
	Creatinine levels (GCST90025946)	1	Wald ratio	0.0724	<0.001	<0.001	1.075 (1.041–1.11)
	Glomerular filtration rate (GCST003372)	1	Wald ratio	−0.0141	0.040	0.376	0.986 (0.973–0.999)
	Glomerular filtration rate (GCST90026654)	1	Wald ratio	−0.0075	<0.001	<0.001	0.993 (0.99–0.995)
	Glomerular filtration rate (GCST90103634)	1	Wald ratio	−0.0092	<0.001	<0.001	0.991 (0.987–0.995)
	Urinary albumin excretion (GCST006586)	1	Wald ratio	−0.0388	0.003	0.101	0.962 (0.938–0.987)
	Urinary albumin excretion (GCST008794)	1	Wald ratio	−0.0435	0.004	0.075	0.957 (0.929–0.987)
	Urinary albumin excretion (GCST009640)	1	Wald ratio	−0.0542	<0.001	0.022	0.947 (0.919–0.977)
PDIA3	Creatinine levels (GCST90014001)	1	Wald ratio	0.0483	<0.001	<0.001	1.049 (1.025–1.075)
	Creatinine levels (GCST90018979)	1	Wald ratio	0.0430	<0.001	0.002	1.044 (1.021–1.068)
	Creatinine levels (GCST90025946)	1	Wald ratio	0.0496	<0.001	0.004	1.051 (1.022–1.08)
	Glomerular filtration rate (GCST003372)	1	Wald ratio	−0.0057	0.368	0.754	0.994 (0.982–1.007)
	Glomerular filtration rate (GCST90026654)	1	Wald ratio	−0.0070	<0.001	<0.001	0.993 (0.99–0.996)
	Glomerular filtration rate (GCST90103634)	1	Wald ratio	−0.0063	<0.001	0.007	0.994 (0.99–0.997)
	Urinary albumin excretion (GCST006586)	1	Wald ratio	−0.0325	0.006	0.135	0.968 (0.946–0.991)
	Urinary albumin excretion (GCST008794)	1	Wald ratio	−0.0544	<0.001	0.006	0.947 (0.921–0.974)
	Urinary albumin excretion (GCST009640)	1	Wald ratio	−0.0399	0.006	0.085	0.961 (0.934–0.989)
PPTC7	Creatinine levels (GCST90014001)	1	Wald ratio	−0.0236	0.007	0.034	0.977 (0.96–0.993)
	Creatinine levels (GCST90018979)	1	Wald ratio	−0.0306	0.001	0.007	0.97 (0.952–0.988)
	Creatinine levels (GCST90025946)	1	Wald ratio	−0.0248	0.027	0.098	0.975 (0.954–0.997)
	Glomerular filtration rate (GCST003372)	1	Wald ratio	0.0069	0.296	0.745	1.007 (0.994–1.02)
	Glomerular filtration rate (GCST90026654)	1	Wald ratio	0.0040	<0.001	0.004	1.004 (1.002–1.006)
	Glomerular filtration rate (GCST90103634)	1	Wald ratio	0.0042	0.005	0.028	1.004 (1.001–1.007)
	Urinary albumin excretion (GCST006586)	1	Wald ratio	0.0207	0.013	0.176	1.021 (1.004–1.038)
	Urinary albumin excretion (GCST008794)	1	Wald ratio	0.0291	0.004	0.068	1.03 (1.01–1.05)
	Urinary albumin excretion (GCST009640)	1	Wald ratio	0.0306	0.003	0.050	1.031 (1.011–1.052)
PTPN9	Creatinine levels (GCST90018979)	1	Wald ratio	0.1541	<0.001	<0.001	1.167 (1.12–1.215)
	Creatinine levels (GCST90025946)	1	Wald ratio	0.2723	<0.001	<0.001	1.313 (1.246–1.383)
	Glomerular filtration rate (GCST003372)	1	Wald ratio	−0.0232	0.046	0.393	0.977 (0.955–1)
	Glomerular filtration rate (GCST90026654)	1	Wald ratio	−0.0317	<0.001	<0.001	0.969 (0.963–0.974)
	Glomerular filtration rate (GCST90103634)	1	Wald ratio	−0.0359	<0.001	<0.001	0.965 (0.958–0.971)
	Urinary albumin excretion (GCST006586)	1	Wald ratio	−0.0668	0.003	0.101	0.935 (0.895–0.977)
	Urinary albumin excretion (GCST008794)	1	Wald ratio	−0.0854	0.001	0.036	0.918 (0.871–0.967)
	Urinary albumin excretion (GCST009640)	1	Wald ratio	−0.0950	<0.001	0.017	0.909 (0.863–0.958)
RDH10	Creatinine levels (GCST90014001)	2	Inverse variance weighted	−0.0335	0.003	0.040	0.967 (0.946–0.988)
	Creatinine levels (GCST90018979)	2	Inverse variance weighted	−0.0277	0.007	0.094	0.973 (0.953–0.993)
	Creatinine levels (GCST90025946)	2	Inverse variance weighted	−0.0405	0.001	0.015	0.96 (0.937–0.985)
	Glomerular filtration rate (GCST003372)	2	Inverse variance weighted	0.0036	0.529	0.988	1.004 (0.993–1.015)
	Glomerular filtration rate (GCST90026654)	2	Inverse variance weighted	0.0037	0.011	0.100	1.004 (1.001–1.007)
	Glomerular filtration rate (GCST90103634)	2	Inverse variance weighted	0.0051	0.001	0.021	1.005 (1.002–1.008)
	Urinary albumin excretion (GCST006586)	2	Inverse variance weighted	0.0373	0.001	0.115	1.038 (1.014–1.062)
	Urinary albumin excretion (GCST008794)	2	Inverse variance weighted	0.0566	<0.001	0.006	1.058 (1.032–1.085)
	Urinary albumin excretion (GCST009640)	2	Inverse variance weighted	0.0473	<0.001	0.043	1.048 (1.022–1.076)
TWF2	Creatinine levels (GCST90014001)	1	Wald ratio	0.1349	<0.001	<0.001	1.144 (1.102–1.188)
	Creatinine levels (GCST90018979)	1	Wald ratio	0.0913	<0.001	<0.001	1.096 (1.059–1.134)
	Creatinine levels (GCST90025946)	1	Wald ratio	0.1696	<0.001	<0.001	1.185 (1.134–1.238)
	Glomerular filtration rate (GCST003372)	1	Wald ratio	−0.0215	0.022	0.279	0.979 (0.961–0.997)
	Glomerular filtration rate (GCST90026654)	1	Wald ratio	−0.0150	<0.001	<0.001	0.985 (0.98–0.99)
	Glomerular filtration rate (GCST90103634)	1	Wald ratio	−0.0185	<0.001	<0.001	0.982 (0.976–0.988)
	Urinary albumin excretion (GCST006586)	1	Wald ratio	−0.0895	<0.001	<0.001	0.914(0.882–0.948)
	Urinary albumin excretion (GCST008794)	1	Wald ratio	−0.1009	<0.001	<0.001	0.904(0.866–0.944)
	Urinary albumin excretion (GCST009640)	1	Wald ratio	−0.1067	<0.001	<0.001	0.899(0.86–0.939)

### 3.4. Microarray expression data validation

**Figure 4.**
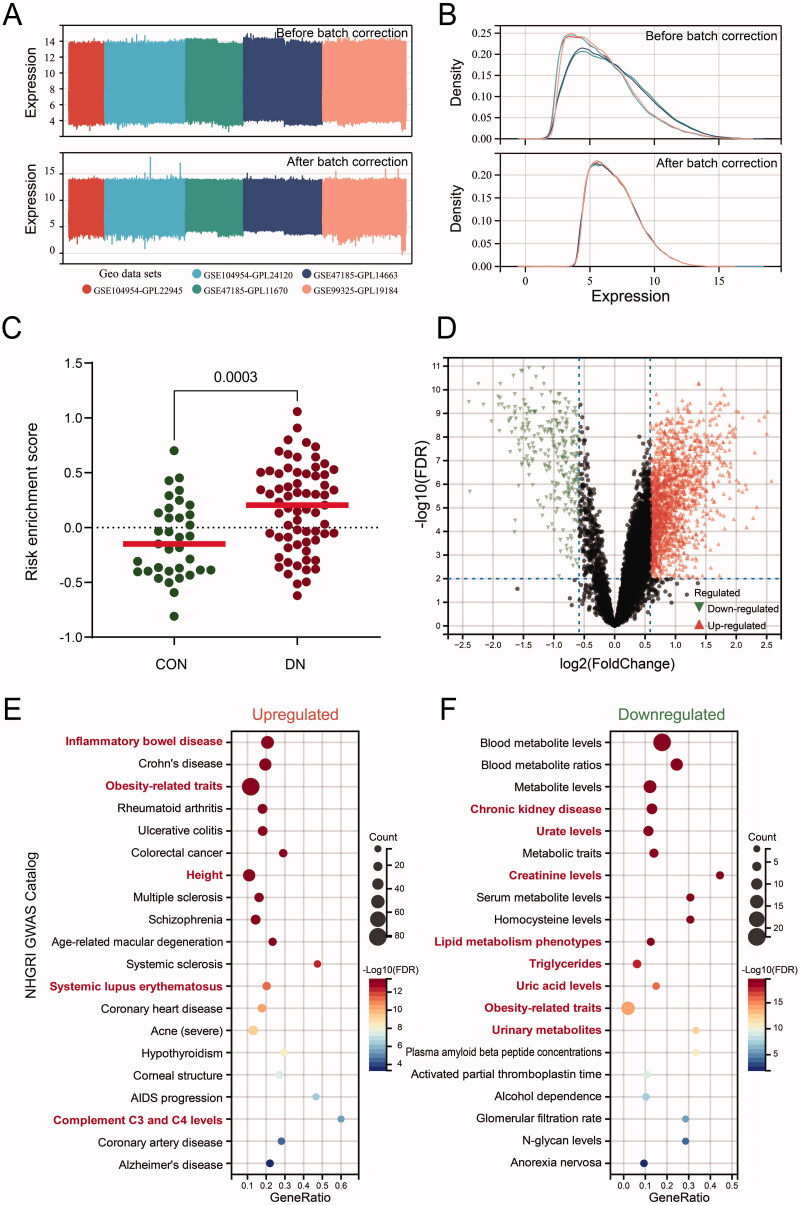
Grouping analysis based on GSVA score according to MR results. (A, B) Expression box plot (left) and density plot (right) of GEO datesets. Above: before adjusting batch effects. Below: after adjusting batch effects. Adjusting batch effects using empirical Bayes method. (C) Statistical results of GSVA score. Risk enrichment score was calculated: risk-gene set score minus resistance-gene set score. (D) Volcano plot of differentially expressed genes in DN samples, grouped by median of risk enrichment score. (E–F) National Human Genome Research Institute (NHGRI) GWAS Catalog enrichment analysis of differentially expressed genes was performed by KOBAS version 3.0. The size of the knots represents the number of enriched genes.

**Figure 5.**
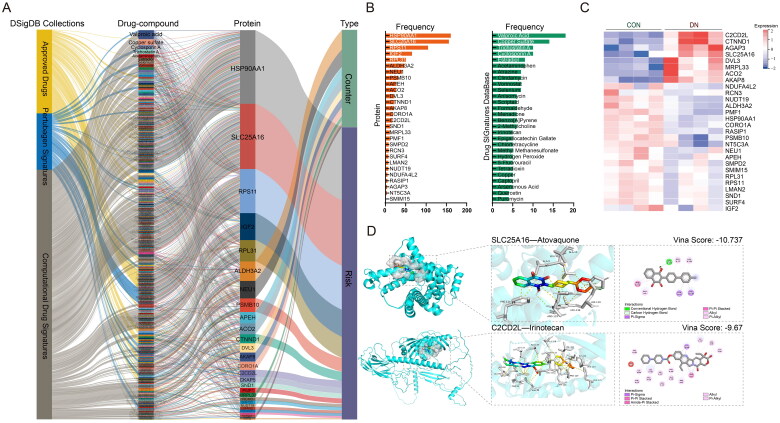
Correlation analysis between drug-component and core proteins. (A) drug-component-target network based on Drug SIGnatures DataBase (https://dsigdb.tanlab.org/DSigDBv1.0/). (B) Statistical results of enriched node frequency. Left: the quantity of drug-component enriched by core proteins. Right: the quantity of core proteins enriched by drug-component. (C) Heat map of abundance values of core proteins based on mass spectrometry. (D) Virtual docking of Atovaquone with SLC25A16 and Irinotecan with C2CD2L was performed by AutoDock Vina.

To further corroborate the core-proteins prediction results of MR analysis, we collected renal biopsy gene expression profile from GEO datasets (GSE104954, GSE47185, and GSE99325). And removed the batch effects from the datasets by using empirical Bayes methods [34] (Figure 4(A, B)). Then the control and DN samples were extracted from the datasets (Supplementary Table S5). The GSVA analysis using risk and resistance dataset was implemented to calculate a risk enrichment score (GSVA_score_risk – GSVA_score_resistance), the result shown discriminated considerable variations in control and DN samples (Figure 4(C) and Supplementary Table S6). DN samples were separated into two groups according to the median of the risk enrichment score. The differential gene expression between high and low score groups were explored *via* limma package (version 3.40.6) (Figure 5(D)). The NHGRI GWAS Catalog analysis was performed to indentify differential genes associated with disease phenotypes in the high risk enrichment score DN group. For the up-regulated differential genes, ‘Inflammatory bowel disease’ and ‘Obesity-related traits’ were the highest enrichment terms (Figure 4(E)). Down-regulated differential genes enrichment analysis revealed several kidney disease terms were enriched, notably ‘Chronic kidney disease’, ‘Urate levels’, ‘Creatinine levels’ and ‘Lipid metabolism phenotypes’ terms (Figure 4(F)). These results suggested that these core-targets selected by MR analysis were significantly associated with DN risk.

### 3.5. Drug-compound–target interaction analysis

The interaction between core-protein and drug-component was shown in Figure 5(A). The annotated drug/compound information was collected *via* Drug SIGnatures DataBase (Supplementary Table S7). ‘Approved Drugs’ represent FDA-approved drug datasets. ‘Perturbagen Signatures’ data originate from Connectivity Map (CMap) database [35]. ‘Computational Drug Signatures’ information extracted from literatures [36]. More than one hundred drugs/compounds interact with HSP90AA1 and SLC25A16, suggesting that their structures may be advantageous for drug design (Figure 5(B)). Valproic acid could affect most core-protein, may be a compound associated with the progression of DN (Figure 5(B)). Considering the compensatory mechanisms of organisms, some risk proteins may be downregulated, and vice versa. In our result, SLC25A16, CTNND1, and C2CD2L in risk set were upregulated, and ALDH3A2, NEU1, APEH, CORO1A, NUDT19, and NDUFA4L2 in resistance set were downregulated (Figure 5(C)). To annotate the functions of these proteins, we conducted enrichment analysis utilizing biophysical interactions of ORFeome-based complexes network (BioPlex version 3.0) and Protein-Protein Interaction Networks (STRING version 12.0) [37,38], found that they are mainly annotated to metabolism, transport, and mitochondrial terms (Supplementary Table S8). To verify their clinical value, we performed molecular docking between these nine core proteins and their paired drug ligands using AutoDock Vina, and found that stable structures can be formed between them (Supplementary Table S9). Such as SLC25A16- Atovaquone and C2CD2L- Irinotecan (Figure 5(D)). These nine core-proteins may be significantly associated with DN mitochondrial dysfunction and were potential targets for anti-DN drugs design.

## 4. Discussion

To our knowledge, there are no similar results published in the literature for DN mitochondrial proteomes combined with MR analysis. We performed DN mitochondrial proteomes by mass spectrometry, screened core proteins *via* MR analysis, and validated them in renal microarray expression datasets. Finally, nine proteins (SLC25A16, CTNND1, C2CD2L, ALDH3A2, NEU1, APEH, CORO1A, NUDT19, and NDUFA4L2) as potential drug targets for DN were identified. This finding provides a new direction for future drug design in DN therapeutics.

When UACR ≥ 30 mg/g and eGFR < 60 mL/min/1.73 m^2^ lasts for more than 3 months, excluding other factors CKD, the diabetes patients can be diagnosed as DN [39]. Nearly all the types of renal cells, including podocytes, mesangial cells, endothelial cells, tubular epithelial cells, interstitial fibroblasts, and vascular endothelial cells, are affected by hyperglycemia, which promotes mesangial proliferation, hypertension, glomerulosclerosis, and renal fibrosis [40,41]. These factors make DN the leading cause of ESRD that requires renal replacement therapies. Based on this, considerable drug development research has been conducted in recent years. The most well-known representative drugs among them are sodium-dependent glucose transporters 2 (SGLT2) inhibitors, mineralocorticoid receptor antagonists, and glucagon-like peptide-1 (GLP-1) receptor agonist. SGLT2 is responsible for 90% of glucose reabsorption in proximal tubule [21], mineralocorticoid receptor plays a vital role in water and electrolyte balance [42], and GLP-1 participates in blood glucose regulation by activating GLP-1 receptors to increase insulin secretion and decrease glucagon secretion [43]. They have been widely used in the treatment of DN at distinct phases. However, these drugs can only decelerate the progressive of DN. Once diagnosed, most DN patients seem to be progressing toward ESRD with an unstoppable trend [11,44,45]. This indicates that we still do not fully understand the mechanism of DN progression, and new therapeutic targets still need to be discovered and studied.

In the early stage of DN, the delivery of metabolic substrates for ATP production in renal cells, such as fatty acids and oxygen, is altered due to diabetes. The mitochondrial adaptation of metabolic fuel sources to meet ATP demands results in excessive generation of reactive oxygen species and disruption of cellular signaling, which is reported to accelerate the progression of DN [46–48]. In this study, we analyzed proteins changes in DN renal mitochondria using mass spectrometry and identified many differentially expressed proteins (Figure 1). However, it is difficult to distinguish which of them play a core role in the progression of DN. MR is one of the emerging approaches to strengthen causal inference based on the IV method. Increasing evidence suggests that MR studies have broad potential in improving drug development efficiency and reducing the risk of expensive late stage failures. Previous MR analysis in drug screening studies have primarily concentrated on other diseases, such as rheumatism, cancer, cardiopathy, and neurodegenerative diseases [49–52]. And there are few reports on the combination of subcellular organelle proteomics and MR analysis in DN research.

In this study, given the promising results of MR analysis in screening novel drug targets, we performed MR analysis between differentially expressed mitochondrial proteins and DN risk factors (eGFR, urinary albumin excretion and serum creatinine level), to screen potential therapeutic targets for DN. The datasets we collect for MR analysis are sourced from various independent studies, including the CKDGen, UK Biobank, FinnGen database, and others. To address potential sample overlap in MR analysis, only when these differentially expressed proteins can be enriched in at least six datasets can they be considered candidate targets. After MR analysis, 28 candidate core proteins were preliminarily identified based on the above strategy (Figures 2–3). Through renal microarray expression datasets validation, we find that these candidate proteins, located in the mitochondrion, exhibit close associations with obesity, metabolism, and inflammation (Figure 4). According to the literature, obesity and metabolic abnormalities have made DN the leading cause of CKD and ESRD [53,54]. Obesity can lead to various structural and metabolic changes in the kidneys, with the metabolic alterations triggering the activation of inflammatory pathways, which in turn causes renal functional and structural injury [54]. The mitochondrial abnormalities are precisely the core of all these mutations. Some mitochondrial proteins have been identified as important targets for kidney disease treatment. Such as SIRT3, localizes in the mitochondrial matrix, can improve the production of energy to against worsening kidney injury [55]. And transgenic expression of PGC-1α in renal tubular mitochondria could improve kidney histology by enhancing fatty acid oxidation [56]. In our study, more renal tubular mitochondrial proteins related to DN were identified by MR analysis. And combining the results of protein abundance in mass spectrometry, nine core proteins (SLC25A16, CTNND1, C2CD2L, ALDH3A2, NEU1, APEH, CORO1A, NUDT19, and NDUFA4L2) were ultimately identified as potential drug targets for DN treatment (Figure 5). Function enrichment analysis suggest that these proteins mainly related to mitochondrial metabolism and substance transport. Changes in the abundance of these proteins may play an important role in the progression of DN by interfering with mitochondrial homeostasis.

Through molecular docking based on the enrichment results of the DSigDB database, we found that most core proteins exhibit promising druggability (Supplementary Table S9). Such as SLC25A16, which is located in the inner membrane and contains three tandemly repeated mitochondrial carrier protein domains, MR analysis has demonstrated that its expression level is positively correlated with the risk of DN. Molecular docking shows that SLC25A16 has appreciable binding affinity for multiple ligands, including atovaquone and calmidazolium. Atorvaquinone is a naphthoquinone compound and calmidazolium is widely used as a calmodulin antagonist. Although the role of these ligands in DN has been rarely reported, the implications suggest that SLC25A16 has druggability potential. However, the exact function and substrate of SLC25A16 have not been accurately determined, with only a speculative substrate of coenzyme A has been observed in yeast [57]. Most of the core proteins identified in our study are novel targets in the pathogenesis of DN. Although the specific mechanisms of these targets in DN progression require further research, this study preliminarily demonstrates the potential of combining MR analysis with proteomics to identify novel therapeutic targets for kidney diseases.

There are some limitations in this study. Initially, mitochondria were extracted from mouse animal model, not from renal biopsy samples of DN patients. Using animal models to simulate human diseases is a common research method, but the differences between patients and animal models remain significant. In subsequent studies, mitochondria will be extracted from DN patients in compliance with ethical requirements, and multiple animal models validation will be conducted. Secondly, specificity validation of DN screening proteins in human tissues is insufficient. Investigating whether these proteins play important roles in other mitochondrial-related diseases, such as heart disease, neurodegenerative disorders, cancer, and muscle disease, is also a promising direction for future studies. Additionally, this study primarily presents descriptive results and does not fully explore the exact contributions of the screened core proteins in the pathogenesis of DN. We will delve into their potential mechanisms in future research.

To summarize, this study established a strategy centered on the ‘proteomes-MR’ to identify novel therapeutic targets in large datasets, and investigate their role in DN pathogenesis. Our findings have the potential to improve the analysis of DN mechanisms, clinical diagnosis, and drug development.

## Supplementary Material

Original Image for Fig 1 A_Middle Right.jpeg

Original Image for Fig 1 A_Middle Left.jpeg

outline.jpg

Original Image for Fig 1 A_Lower Right.jpg

Original Image for Fig 1 A_Upper Left.jpeg

Supplementary Tables.xlsx

Original Image for Fig 1 A_Lower Left.jpeg

Original Image for Fig 1 C_Right.tif

Original Image for Fig 1 A_Upper Right.jpeg

Original Image for Fig 1 C_Left.tif

## Data Availability

The data underlying this article will be shared upon reasonable request to the corresponding author.
