# Supplementary figures and images for: Combining mitochondrial proteomes and Mendelian randomization to identify novel therapeutic targets for diabetic nephropathy
Source: Ren Fail. 2025 Mar 24;47(1):2473669. doi: 10.1080/0886022X.2025.2473669 (PMC11934170; doi:10.1080/0886022X.2025.2473669)

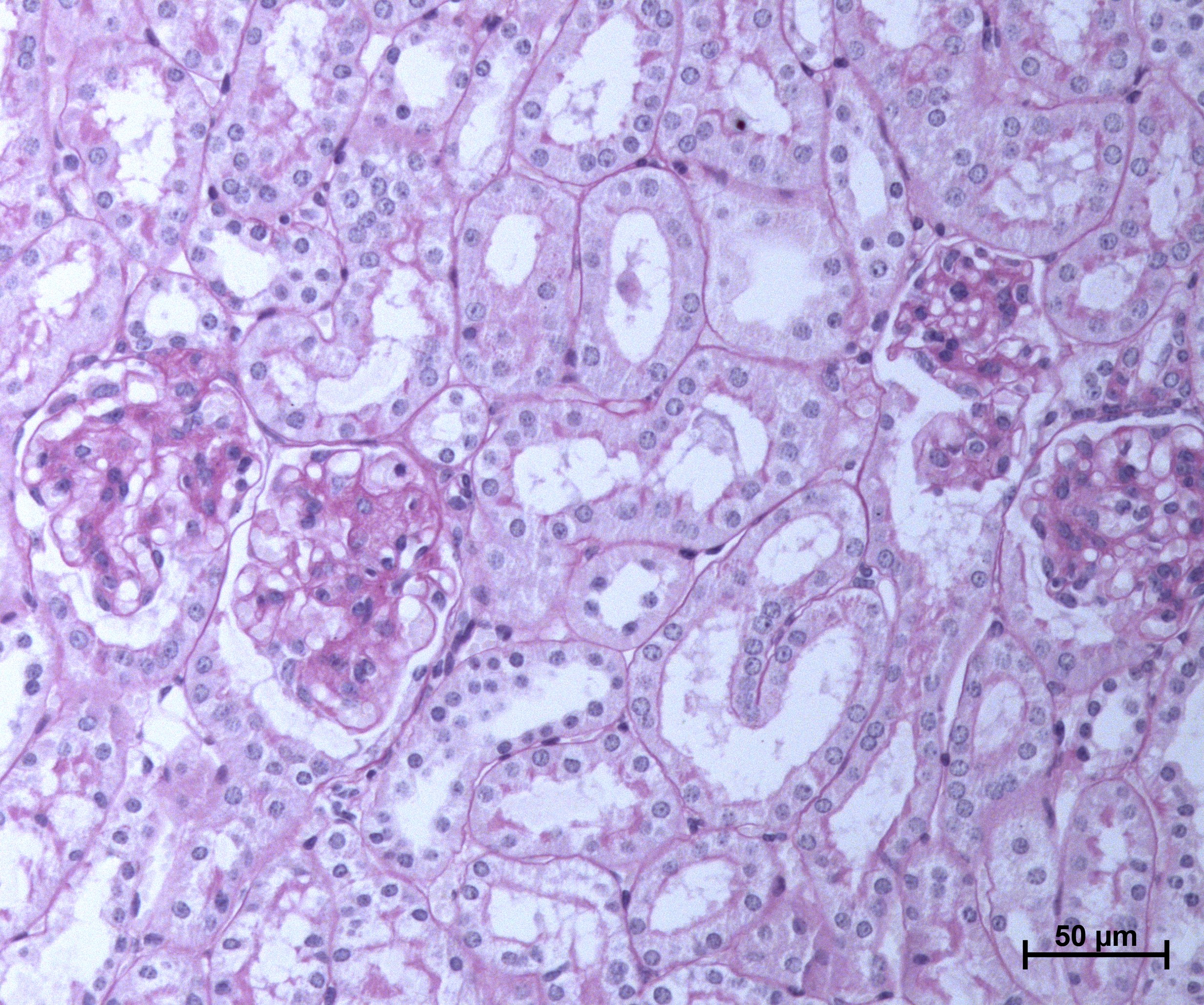

Supplement: Original Image for Fig 1 A_Middle Right.jpeg [file IRNF_A_2473669_SM9078.jpeg]

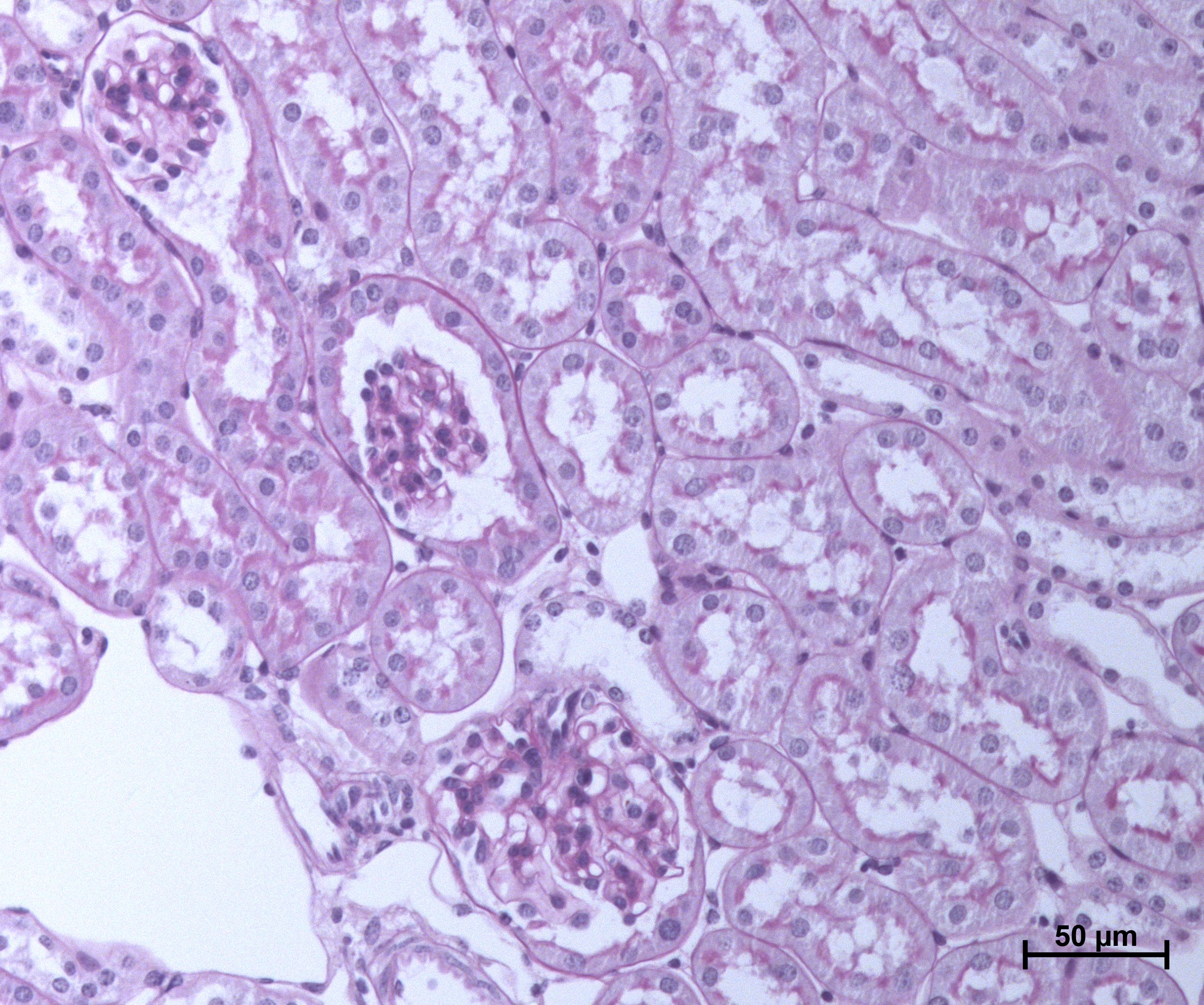

Supplement: Original Image for Fig 1 A_Middle Left.jpeg [file IRNF_A_2473669_SM9077.jpeg]

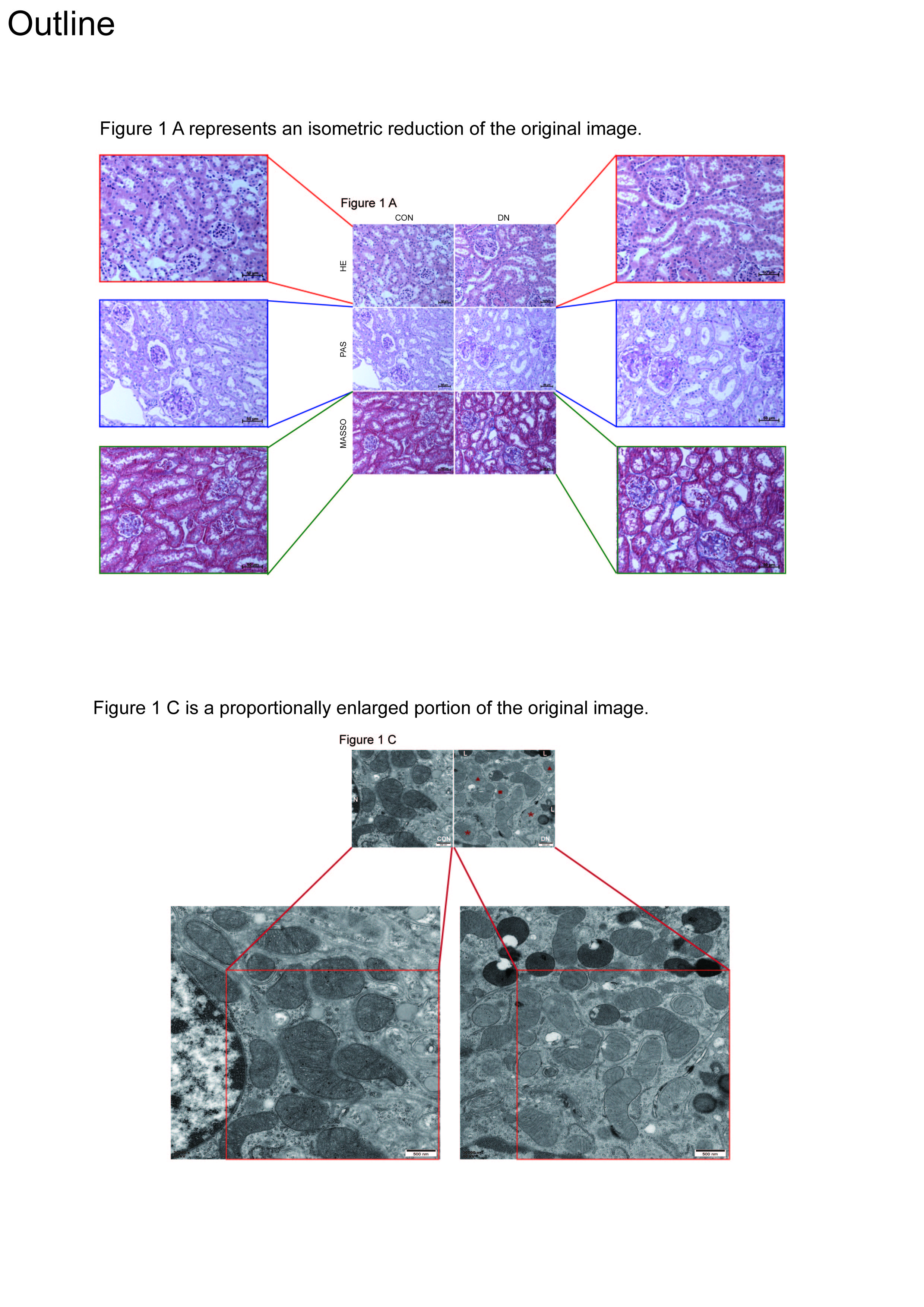

Supplement: outline.jpg [file IRNF_A_2473669_SM9076.jpg]

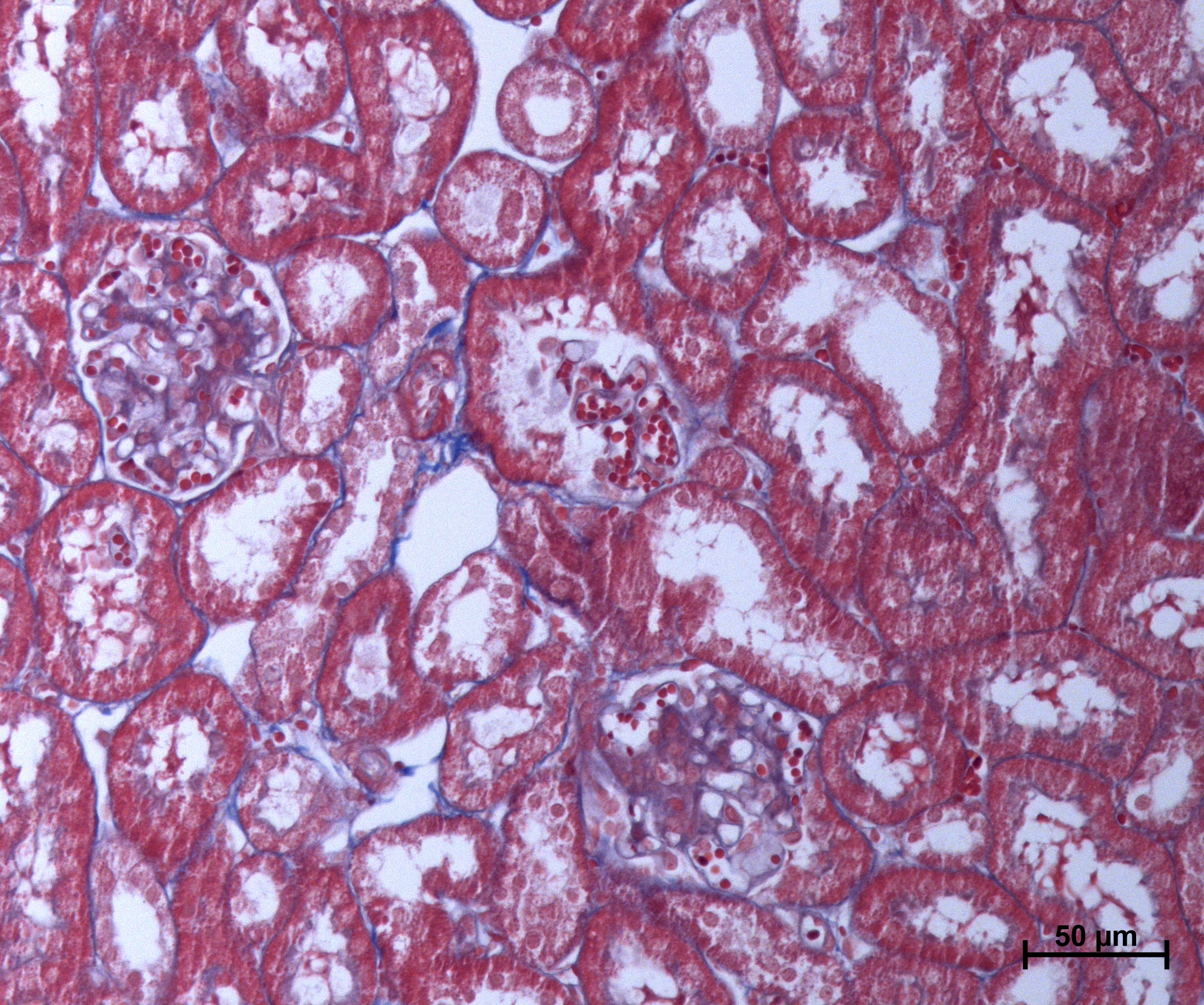

Supplement: Original Image for Fig 1 A_Lower Right.jpg [file IRNF_A_2473669_SM9075.jpg]

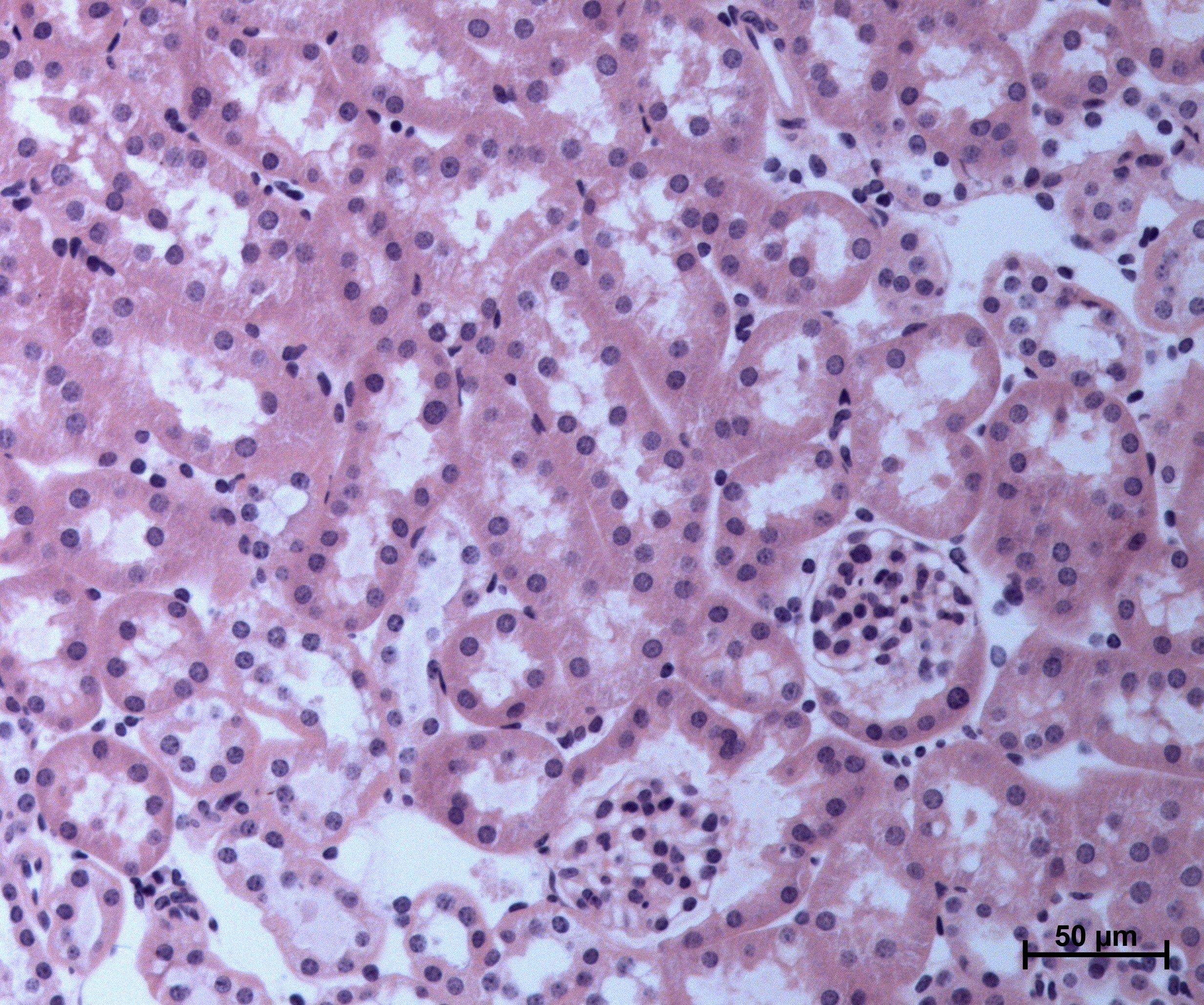

Supplement: Original Image for Fig 1 A_Upper Left.jpeg [file IRNF_A_2473669_SM9074.jpeg]

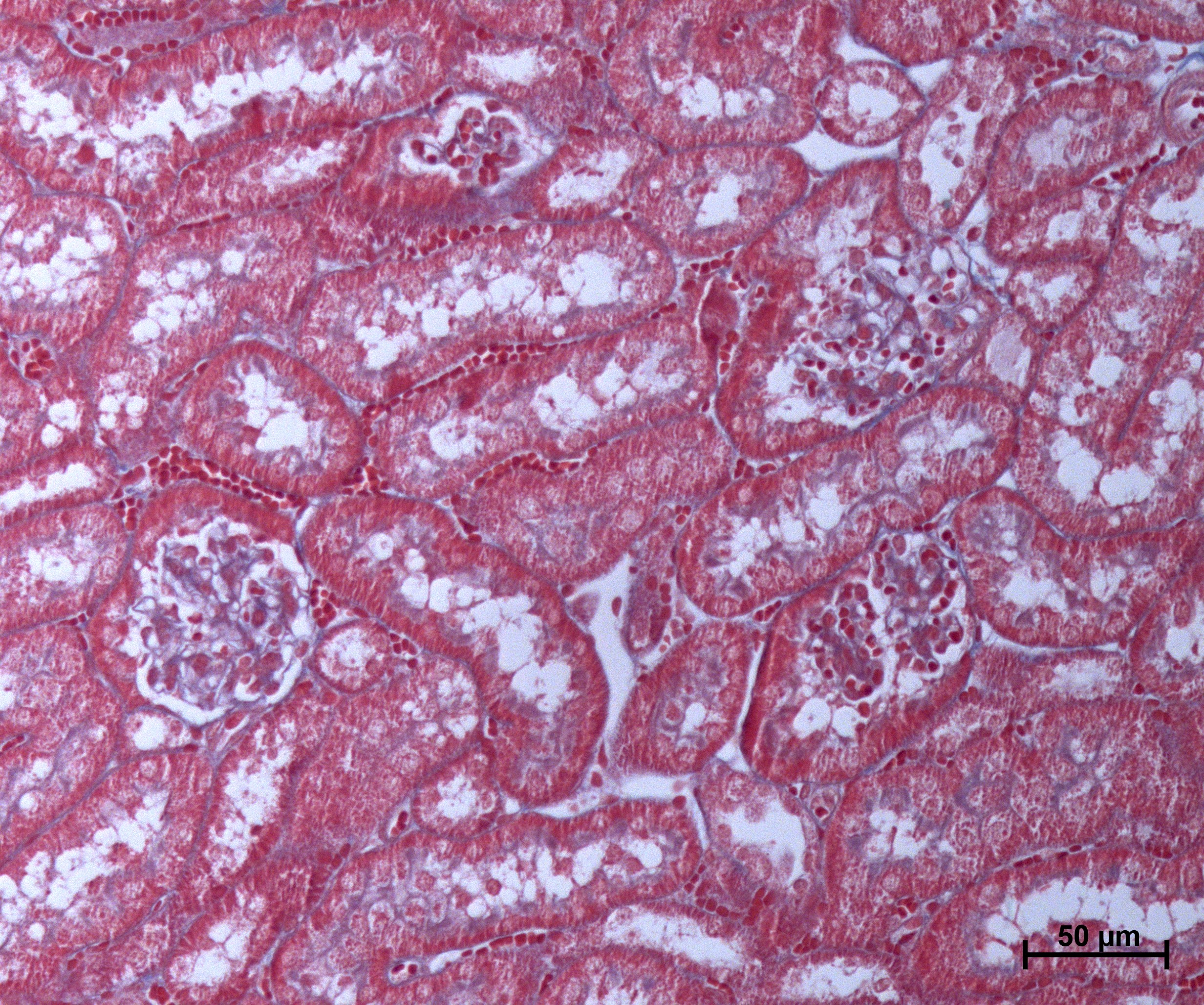

Supplement: Original Image for Fig 1 A_Lower Left.jpeg [file IRNF_A_2473669_SM9072.jpeg]

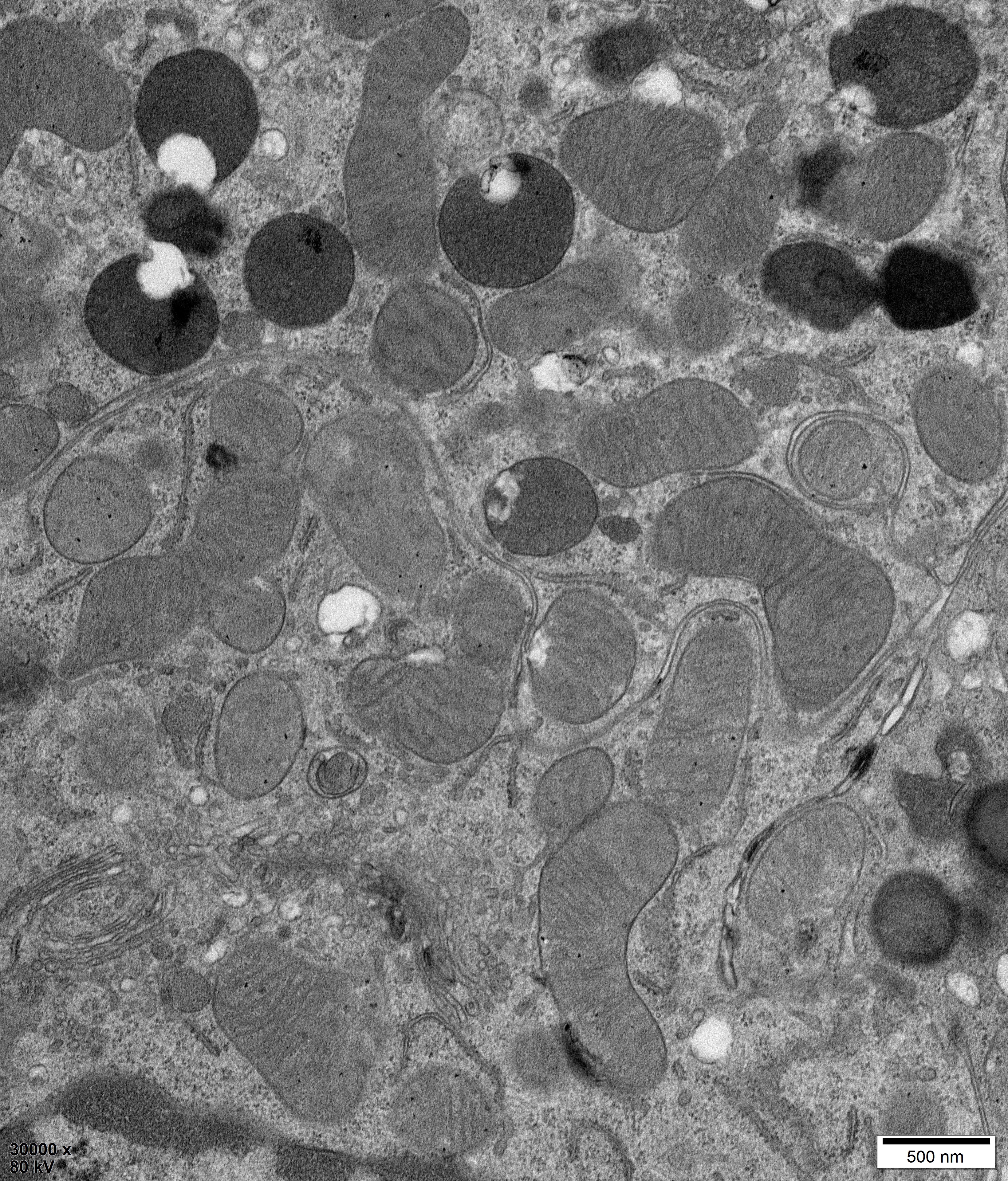

Supplement: Original Image for Fig 1 C_Right.tif [file IRNF_A_2473669_SM9071.tif]

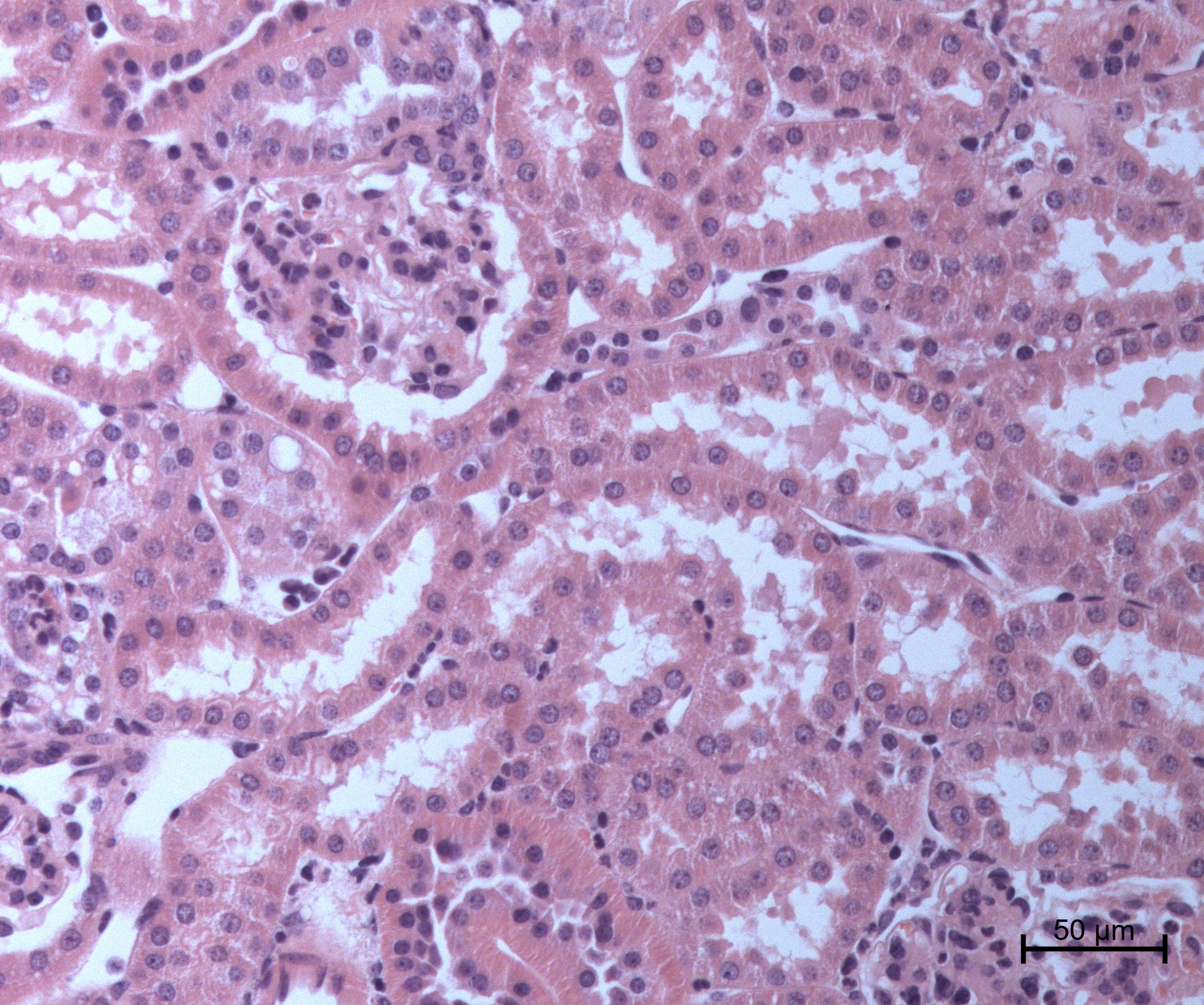

Supplement: Original Image for Fig 1 A_Upper Right.jpeg [file IRNF_A_2473669_SM9070.jpeg]

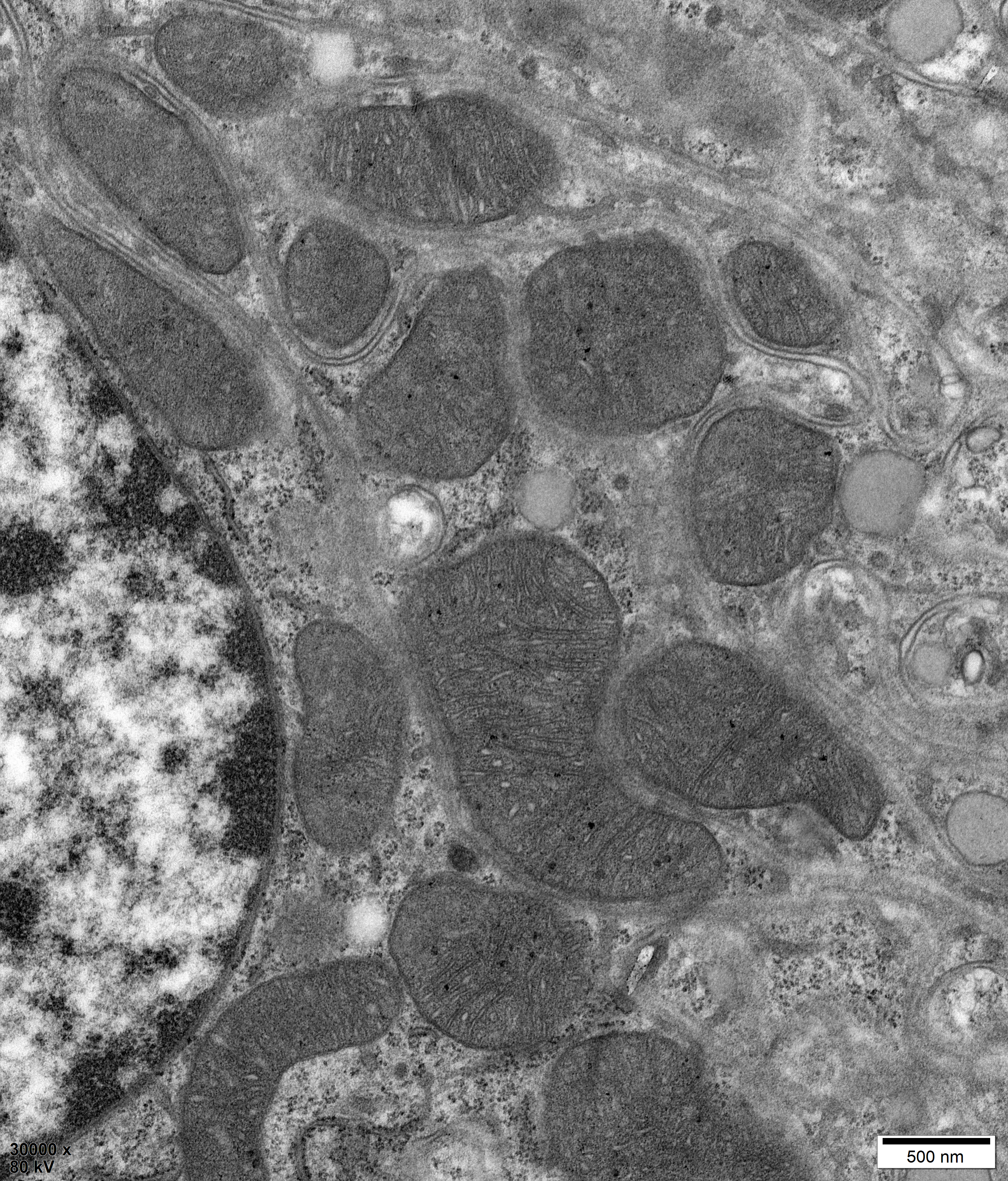

Supplement: Original Image for Fig 1 C_Left.tif [file IRNF_A_2473669_SM9069.tif]
